# Guest‐Modulated Circularly Polarized Luminescence by Ligand‐to‐Ligand Chirality Transfer in Heteroleptic Pd^II^ Coordination Cages

**DOI:** 10.1002/anie.202205725

**Published:** 2022-07-04

**Authors:** Kai Wu, Jacopo Tessarolo, Ananya Baksi, Guido H. Clever

**Affiliations:** ^1^ Department of Chemistry and Chemical Biology TU Dortmund University Otto Hahn Str. 6 44227 Dortmund Germany; ^2^ Department of Chemistry University of Cambridge Lensfield Road Cambridge CB2 1EW UK

**Keywords:** Chirality Transfer, Circularly Polarized Luminescence, Coordination Cages, Host–Guest Chemistry, Supramolecular Chemistry

## Abstract

Multicomponent metallo‐supramolecular assembly allows the rational combination of different building blocks. Discrete multifunctional hosts with an accessible cavity can be prepared in a non‐statistical fashion. We employ our shape‐complementary assembly (SCA) method to achieve for the first time integrative self‐sorting of heteroleptic Pd^II^ cages showing guest‐tunable circularly polarized luminescence (CPL). An enantiopure helicene‐based ligand (**M** or **P** configuration) is coupled with a non‐chiral emissive fluorenone‐based ligand (**A** or **B**) to form a series of Pd_2_L_2_L′_2_ assemblies. The modular strategy allows to impart the chiral information of the helicenes to the overall supramolecular system, resulting in CPL from the non‐chiral component. Guest binding results in a 4‐fold increase of CPL intensity. The principle offers potential to generate libraries of multifunctional materials with applications in molecular recognition, enantioselective photo‐redox catalysis and information processing.

## Graphical abstract

Integrative self‐assembly of Pd^II^ cations with an enantiopure helicene‐based ligand and non‐chiral but emissive fluorenone ligands yields a series of Pd_2_L_2_L′_2_ heteroleptic coordination cages, showing circularly polarized luminescence (CPL) from the non‐chiral moiety. Modulation of the chiroptical properties is achieved by ligand modification or guest binding, achieving an up to 4‐fold enhancement of CPL intensity.

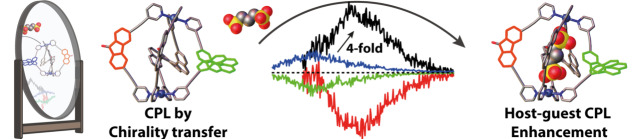

## Introduction

Metal‐mediated self‐assembly of discrete architectures, possessing confined and accessible nanosized cavities, is a consolidated area of supramolecular chemistry.[^1^, ^2^, ^3^, ^4^, ^5^] Owing to the precise geometry, directionality, and often dynamic nature of the metal–ligand interaction, it is possible to design compounds with specific sizes, shapes, and a multitude of properties. The field is inspired by the structure and function of enzymes, nature's primordial host systems. In the last decades, researchers developed numerous artificial host systems offering functions such as selective host–guest interaction,^[6]^ catalysis,[^7^, ^8^, ^9^] sensing^[10]^ and molecular transportation,[^11^, ^12^, ^13^] just to name a few. The choice of tailormade building blocks allows to introduce functional moieties such as dyes,[^14^, ^15^] photoswitches,[^16^, ^17^, ^18^, ^19^] redox centers,[^20^, ^21^, ^22^, ^23^, ^24^] luminophores[^25^, ^26^, ^27^, ^28^, ^29^] or chiral groups.[^30^, ^31^, ^32^, ^33^, ^34^, ^35^, ^36^, ^37^] This can lead to emergent properties, for instance, the introduction of photoswitches^[38]^ has been used to trigger topological rearrangements or guest binding and release.[^19^, ^39^, ^40^] Dynamic helical structures, coupled with dyes or luminophores, have been used to perform chiroptical detection of chiral guests by circular dichroism (CD) or circularly polarized luminescence (CPL) spectroscopy.[^15^, ^41^, ^42^, ^43^]

However, the vast majority of the reported compounds are based on only one type of ligand per assembly, limiting the possibilities to combine several properties and achieve multifunctional supramolecular hosts. While mixing different ligands of similar size and shape can in principle lead to heteroleptic systems, this often results in a statistical mixture of all the possible constituents. Hence, such an approach suffers from a lack of control over stoichiometry and stereochemistry, and complicates the examination and definition of clear structure‐function relationships. To overcome this, in the last few years, several rational strategies to exclusively obtain heteroleptic cages (i.e. species comprising multiple differentiable ligands) in a non‐statistical fashion have been developed.^[44]^ Such strategies include, but are not limited to, coordination sphere engineering (CSE),[^45^, ^46^] charge‐separation,[^47^, ^48^] backbone‐centered steric hindrance,^[49]^ non‐symmetric ligands,[^50^, ^51^] and shape‐complementary assembly (SCA). The latter has been proven as a versatile approach, allowing to achieve heteroleptic supramolecules of different sizes and shapes,[^52^, ^53^, ^54^, ^55^] complex multicavity structures,^[56]^ or cage‐based vesicles.^[57]^ Despite the introduction of these strategies, along with the report of numerous novel structures, examples of multifunctional coordination cages where new properties result from the synergistic interplay of the different building blocks, remain scarce.[^48^, ^57^, ^58^, ^59^, ^60^]

**Figure 1:**
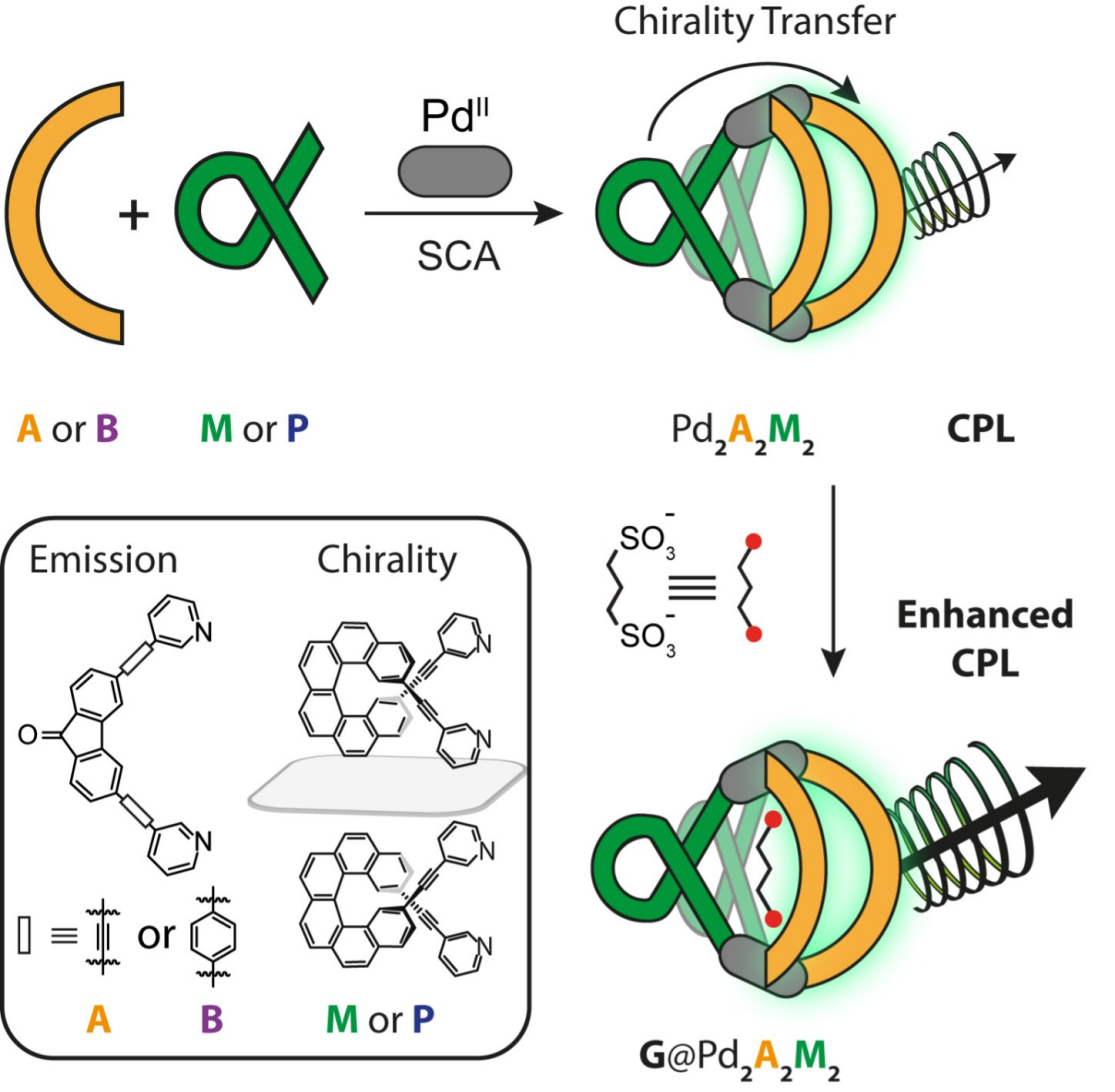
Modular self‐assembly of CPL‐emitting multifunctional cages and guest‐induced tuning of chiroptical signal (ligand structures shown in the box).

In this work we report the self‐assembly of a series of multifunctional Pd_2_L_2_L′_2_ heteroleptic cages showing chiroptical properties deriving from cooperative effects between all of the constituents. The heteroleptic cages are self‐assembled from a banana‐shaped bis‐pyridyl ligand having a fluorenone‐backbone with emissive properties (namely ligands **A** and **B**), a helicene‐based homochiral ligand (**M** or **P** enantiomers) and Pd^II^ cations. Remarkably, the overall properties of the system derive from the combination of the three building blocks. The Pd^II^ metal centers act as structural nodes to assemble a host with an inner cavity large enough to host a small anionic guest. At the same time, the homochiral ligands impart a twist to the overall structure, transferring their chiral information and resulting in CPL emission from the achiral fluorophore‐based ligands (Figure 1). Moreover, the reported Pd_2_L_2_L′_2_ heteroleptic cages are able to bind an aliphatic bis‐sulfonate guest, resulting in pronounced bathochromic shift of the emission and a strong CPL enhancement, providing a unique strategy for probing molecular recognition processes.

## Results and Discussion

### Synthesis and Host–Guest Complex Characterization

Enantiomerically pure helicene‐based ligands, in **M** and **P** configuration, were synthesized as reported by us before.^[32]^ Assembled with Pd^II^ in DMSO, such ligands form Pd_2_L_4_ lantern‐shaped cages, able to bind anionic guest molecules of different sizes and shapes, resulting in modulation of the system's CD signal. Regarding the luminescent ligands, we recently showed that the emission properties of a 2,7‐fluorenone‐based ligand were maintained when self‐assembled with Pd^II^ cations in both homo‐ and heteroleptic assemblies.^[49]^ Moreover, we reported how a 3,6‐fluorenone‐based ligand can form lantern‐shaped Pd_2_L_2_L′_2_ cages according to the shape‐complementarity strategy.[^20^, ^61^] Hence, we decided to use already reported ligand **A** and design a further variation, carrying the same backbone and donor groups, but more rigid and longer 1,4‐phenyl linkers, giving ligand **B** (synthetic details in the Supporting Information).

As mentioned above, the self‐assembly of ligand **M** with metal salt [Pd(CH_3_CN)_4_](BF_4_)_2_ leads to cage Pd_2_ **M**_4_ whose solid state structure was not yet reported. Here, crystals suitable for X‐ray structure analysis were obtained by slow vapor diffusion of ethyl ether into an acetonitrile solution. Homochiral cage Pd_2_ **M**_4_ crystallizes in *P*1 space group, with the asymmetric unit containing one cage molecule (see Figure 3a).^[62]^ The solid‐state structure shows a Pd⋅⋅⋅Pd distance of 8.82 Å, significantly smaller compared to the distance of 11.59 Å measured from a DFT‐calculated model (ωb97xd/def2‐SVP, Figure S29), presumably due to packing and counter anion‐based charge‐screening effects in the solid state as compared to the anion‐free, gas‐phase computed model. As prerequisite for such a discrepancy between the computed and observed structure of the lantern‐shaped assembly, we recognized the helicene ligand's rather large structural flexibility, owing to its spring‐like shape. Indeed, one BF_4_^–^ anion was found to occupy the central cavity of the crystallized cage, stabilized by multiple hydrogen bonds to pyridine‐H atoms, apparently driving the observed cage shrinkage along the Pd_2_‐axis by alleviating the Pd^2+^‐Pd^2+^‐repulsion in contrast to the DFT model of the tetracationic species.

**Figure 2:**
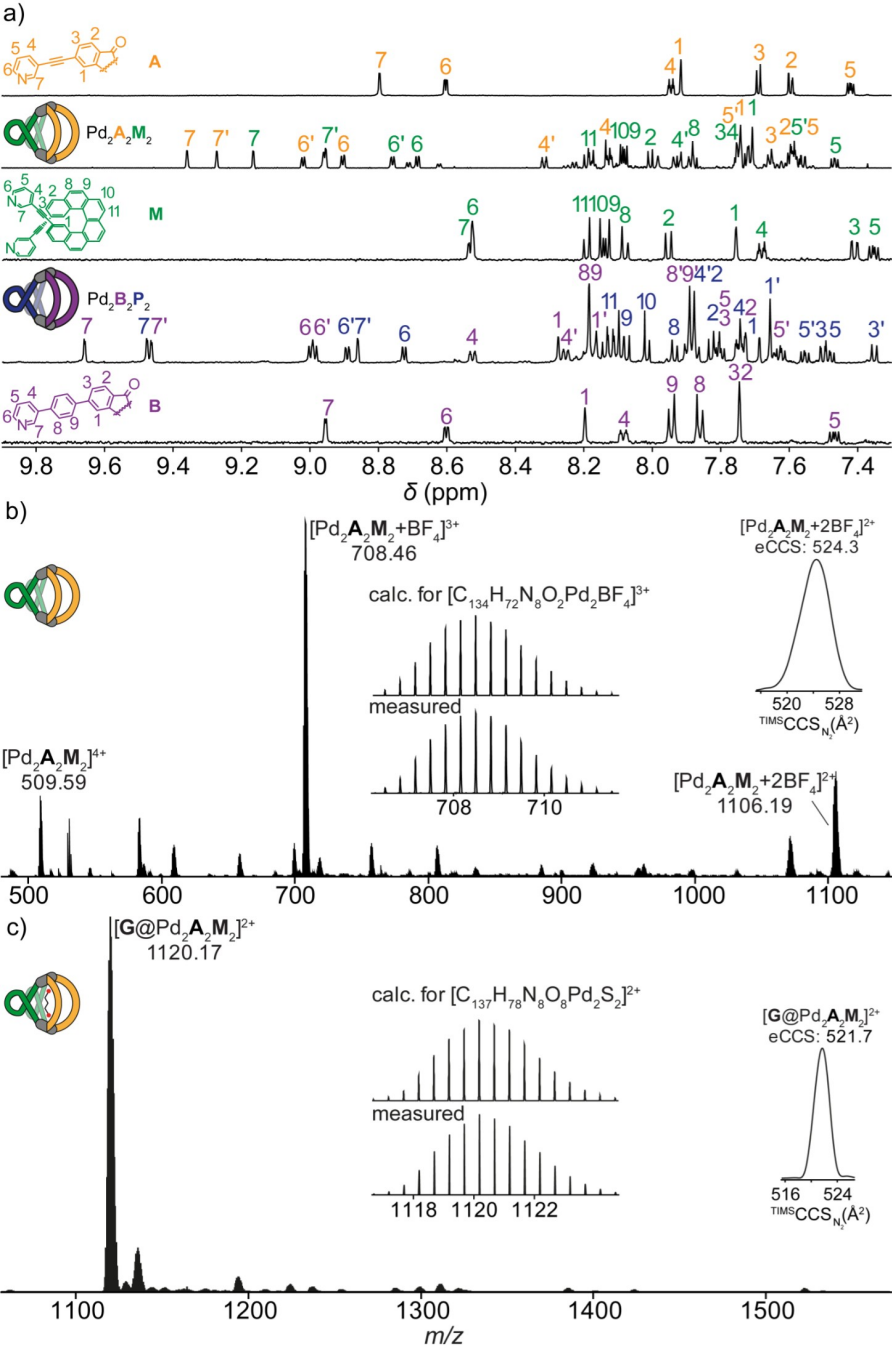
a) From top to bottom, ^1^H NMR spectra (600 MHz, 298 K, CD_3_CN) of ligand **A**, heteroleptic cage Pd_2_ **A**_2_ **M**_2_, ligand **M**, heteroleptic cage Pd_2_ **B**_2_ **P**_2_ and ligand **B**; b) ESI‐MS spectrum of Pd_2_ **A**_2_ **M**_2_, with isotopic pattern for [Pd_2_ **A**_2_ **M**_2_+BF_4_]^3+^; c) ESI‐MS spectrum of **G**@Pd_2_ **A**_2_ **M**_2_, with isotopic pattern for [**G**@Pd_2_ **A**_2_ **M**_2_]^2+^ (ion mobility spectra shown in the insets).

**Figure 3:**
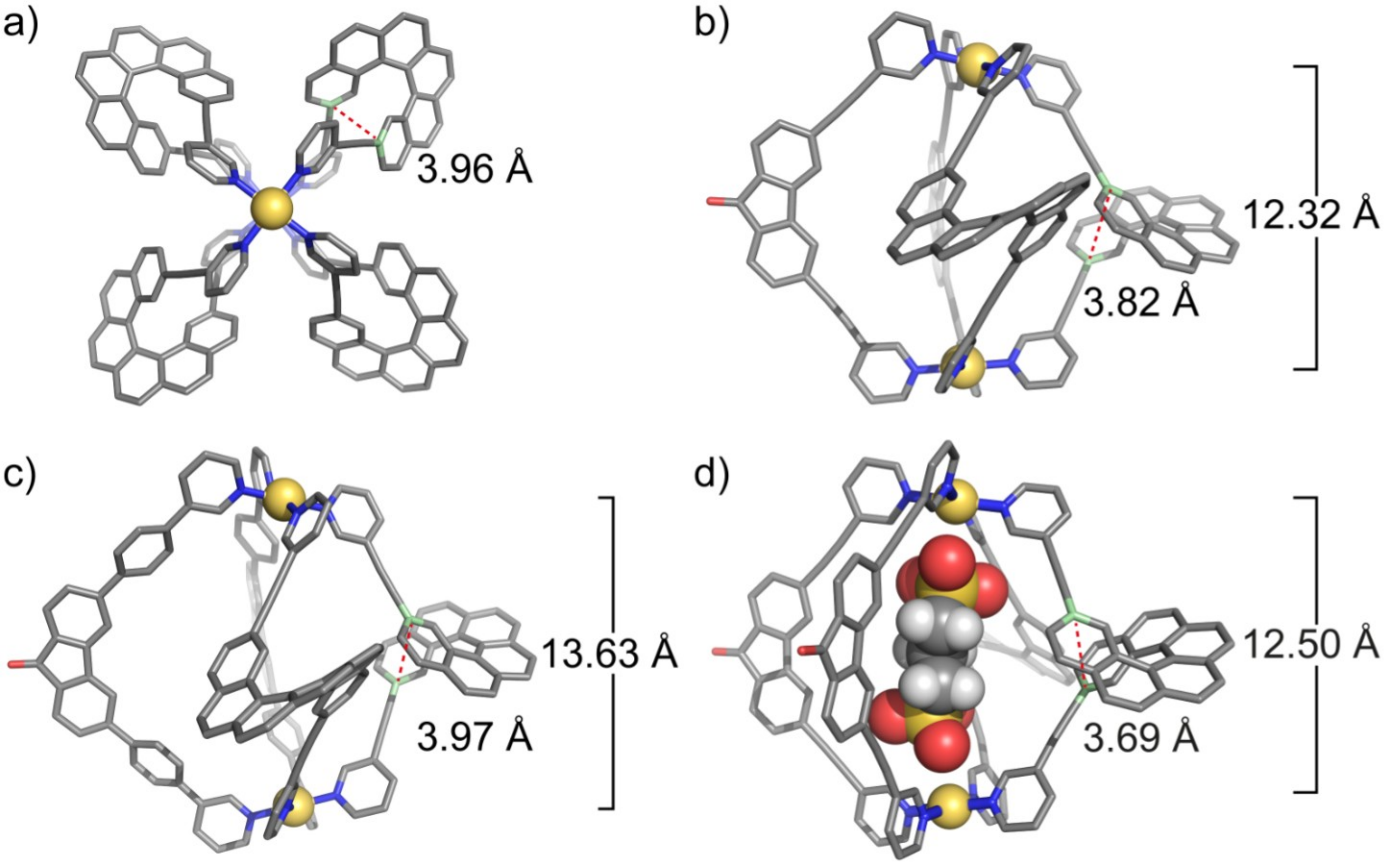
a) X‐ray crystal structure of Pd_2_ **M**_4_, top view; DFT (ωB97XD/def2‐SVP) models of b) Pd_2_ **A**_2_ **M**_2_, c) Pd_2_ **B**_2_ **M**_2_ and d) **G**@Pd_2_ **A**_2_ **M**_2_ with highlight of helical pitch and Pd⋅⋅⋅Pd distances. Color code: Pd yellow, C grey, N blue, O red, S yellow, H white (when not omitted for clarity).

Subsequently, we set out to study the formation of heteroleptic assemblies based on the two types of ligands, utilizing the SCA approach to drive the system to an integrative self‐sorting to give the desired Pd_2_L_2_L′_2_ species. Although the N⋅⋅⋅N distance of fluorenone‐based ligands **A** or **B** is larger than that of ligand **M** or **P**, previous studies indeed showed that helicenes can behave like a mechanical spring, with a modulation of the helical pitch influencing their structure and chiroptical properties.^[32]^ Self‐assembly of Pd^II^, **A**, and **M** in a 1 : 1 : 1 ratio in CD_3_CN at 80 °C for 8 h resulted in a complicated, yet well‐resolved NMR spectrum with two sets of signals, indicating the formation of a discrete heteroleptic assembly (Figure 2a). A ^1^H DOSY experiment confirmed the formation of a single species, with diffusion coefficient *D*=5.85×10^−10^ m^2^ s^−1^, corresponding to a hydrodynamic radius of 1.1 nm, consistent with the expected size for a heteroleptic Pd_2_ **A**_2_ **M**_2_ cage (see below). ^1^H NMR analysis showed significant downfield shifting of the pyridyl protons (Δ*δ*≈0.4 ppm), confirming the coordination of the ligands to the Pd^2+^ metal ions. NOESY correlation between protons H6 and H6′ of the antiparallelly arranged, dissimilar halves of ligand **A**, together with DFT calculations (Supporting Information), support the exclusive formation of the *cis*‐configured heteroleptic cage. In addition, the exclusive formation of the heteroleptic Pd_2_ **A**_2_ **M**_2_ cage was confirmed by high‐resolution electrospray ionization mass spectroscopy (HR‐ESI MS), showing a series of peaks assigned to [Pd_2_ **A**_2_ **M**_2_+*n* BF_4_]^(4−*n*)+^ (*n*=0–2; Figure 2b). Unfortunately, despite numerous trials, all attempts to obtain single crystals for Pd_2_ **A**_2_ **M**_2_ failed. DFT models (ωB97XD/def2‐SVP) were then used to gain more structural insight into the system and to adequately explain the splitting modes observed in the NMR spectra (Figure 3b, Figure S26).

The Pd⋅⋅⋅Pd distance in the model was measured to be 12.32 Å, which is larger than the distance measured in the homoleptic Pd_2_ **M**_4_ DFT‐optimized model (see above). This means that to form the heteroleptic structure, ligand **M** adopts a more stretched conformation to fit its rather rigid counterpart ligand **A**, again emphasizing the spring‐like behaviour of helically twisted helicene. As can be seen from the model, after heteroleptic cage formation, the mirror plane of ligand **A** perpendicular to the plane defined by its backbone is removed due to the absence of a mirror plane in the helicene ligand **M**. Hence, the upper and lower halves of both ligands are no longer equivalent, resulting in the observed splitting of proton signals into two sets. Careful analysis of the splitting pattern, together with COSY and NOESY spectra, allowed us to assign all signals unambiguously (Figure 2a). Similarly, the Pd_2_ **A**_2_ **P**_2_ cage enantiomer was obtained by using ligand **P**, resulting in identical NMR spectra (Figures S5).

The structural flexibility of ligands **M**/**P**, given by the helicenes’ spring‐like behaviour, inspired us to study the assembly of heteroleptic cages using even longer ligand **B** as counterpart, with the aim to further modulate the cavity size. Although, according to DFT models of both cages, the N⋅⋅⋅N distance of ligand **B** is significantly larger than that of **A** (14.82 Å vs. 13.16 Å), when combined with **P**, we obtained a ^1^H NMR spectrum with a similar splitting pattern to what previously observed for Pd_2_ **A**_2_ **M**_2_ (Figure 2a). DOSY analysis again indicates the formation of a single species, with a hydrodynamic radius of 1.3 nm (*D*=5.20×10^−10^ m^2^ s^−1^), supporting the formation of a slightly larger cage structure as compared to Pd_2_ **A**_2_ **M**_2_ (Figures S13). Once more, the opposite enantiomer Pd_2_ **B**_2_ **M**_2_ was also obtained, showing an identical NMR spectrum (Figure S10). HR‐ESI MS again confirmed the formation of a dinuclear heteroleptic cage Pd_2_ **B**_2_ **M**_2_ by showing a series of peaks assigned to [Pd_2_ **B**_2_ **M**_2_+*n* BF_4_]^(4−*n*)+^ (*n*=0–2; Figure S14). The DFT‐optimized model (ωb97xd/def2‐SVP) of Pd_2_ **B**_2_ **M**_2_ gave a Pd⋅⋅⋅Pd distance of 13.63 Å. The longer Pd⋅⋅⋅Pd distance compared with that of Pd_2_ **A**_2_ **M**_2_ suggests that the flexible helicene ligand is able to stretch accordingly to match its counterpart.

Having in hand two sets of heteroleptic cages Pd_2_ **A**_2_(**M**/**P**)_2_ and Pd_2_ **B**_2_(**M**/**P**)_2_ with different cavity sizes, we studied the interaction with an anionic guest, with the goal to subsequently investigate the chiroptical effect of molecular recognition. The host–guest interactions with 1,3‐propane bis‐sulfonate as guest (**G**) were initially investigated by NMR titrations. Upon gradual addition of the guest to Pd_2_ **A**_2_ **M**_2_ in CD_3_CN, inward pointing pyridine protons H7 from both ligands undergo a downfield shift (Δ*δ*≈1.0 ppm), indicating guest binding inside the cavity. In a similar way, addition of **G** to Pd_2_ **B**_2_ **M**_2_ also results in guest encapsulation with slow‐exchange dynamics. Upon addition of 0.4 equiv guest, both empty cage and host–guest complex are distinguishable, while addition of 1.0 equiv guest results in a single set of ^1^H NMR signals, indicating the formation of [**G**@Pd_2_ **B**_2_ **M**_2_]. Unfortunately, determination of association constants was hampered by borderline solubility and apparent aggregation (onset of precipitation>1 equiv guest), leading to rather noisy NMR traces. Further confirmation comes from HR‐ESI‐MS studies, where it was possible to detect the exclusive formation of [**G**@Pd_2_ **A**_2_ **M**_2_]^2+^ (Figure 2c), as well as the analogous [**G**@Pd_2_ **B**_2_ **M**_2_]^2+^ species (Figure S20). Trapped ion mobility spectrometry (ESI‐TIMS‐TOF) for the 2+ peaks of both empty host [2BF_4_+Pd_2_ **A**_2_ **M**_2_]^2+^ and host–guest complex [**G**@Pd_2_ **A**_2_ **M**_2_]^2+^ yielded collisional cross sections (CCS) of 524.3 Å^2^ and 521.7 Å^2^, respectively, confirming an inside binding mode and showing a slight contraction of the host–guest complex (Figure 2b, c insets). An analogous trend is observed for the host and host–guest species with ligand **B**, yielding CCS values of 556.1 Å^2^ and 550.4 Å^2^, respectively (Figure S25). A comparison of DFT‐optimized models of the empty host and host–guest complex proved helpful to understand this behaviour (Figure S28), as the Pd⋅⋅⋅Pd distance in [**G**@Pd_2_ **B**_2_ **M**_2_]^2+^ is indeed smaller than in the empty cage, consistent with the CCS measurement and explainable by the propensity of the dianionic guest to pull the cage's Pd^II^‐faces slightly together.

### Photophysical Characterization

Having the multifunctional heteroleptic cages and host–guest complexes in hand, we investigated their (chir)optical properties. All employed ligands are based on well‐known chromophores. Both ligands **A** and **M**/**P** present similar absorption spectra with absorption bands in the range of 200–400 nm. Specifically, **M**/**P** is characterized by a band centred at 292 nm and a broad shoulder in the range 325–400 nm, while ligand **A** presents two bands at 285 and 300 nm, and a broad band between 325 and 375 nm (Figure S21a). Upon Pd^II^ coordination, the absorption bands of the homoleptic cages Pd_2_(**M**/**P**)_4_ are red shifted, while for Pd_2_ **A**_4_ the spectrum resembles the one of the free ligands, with slight variations in the relative intensity of the different bands (Figure S21b).

**Figure 4:**
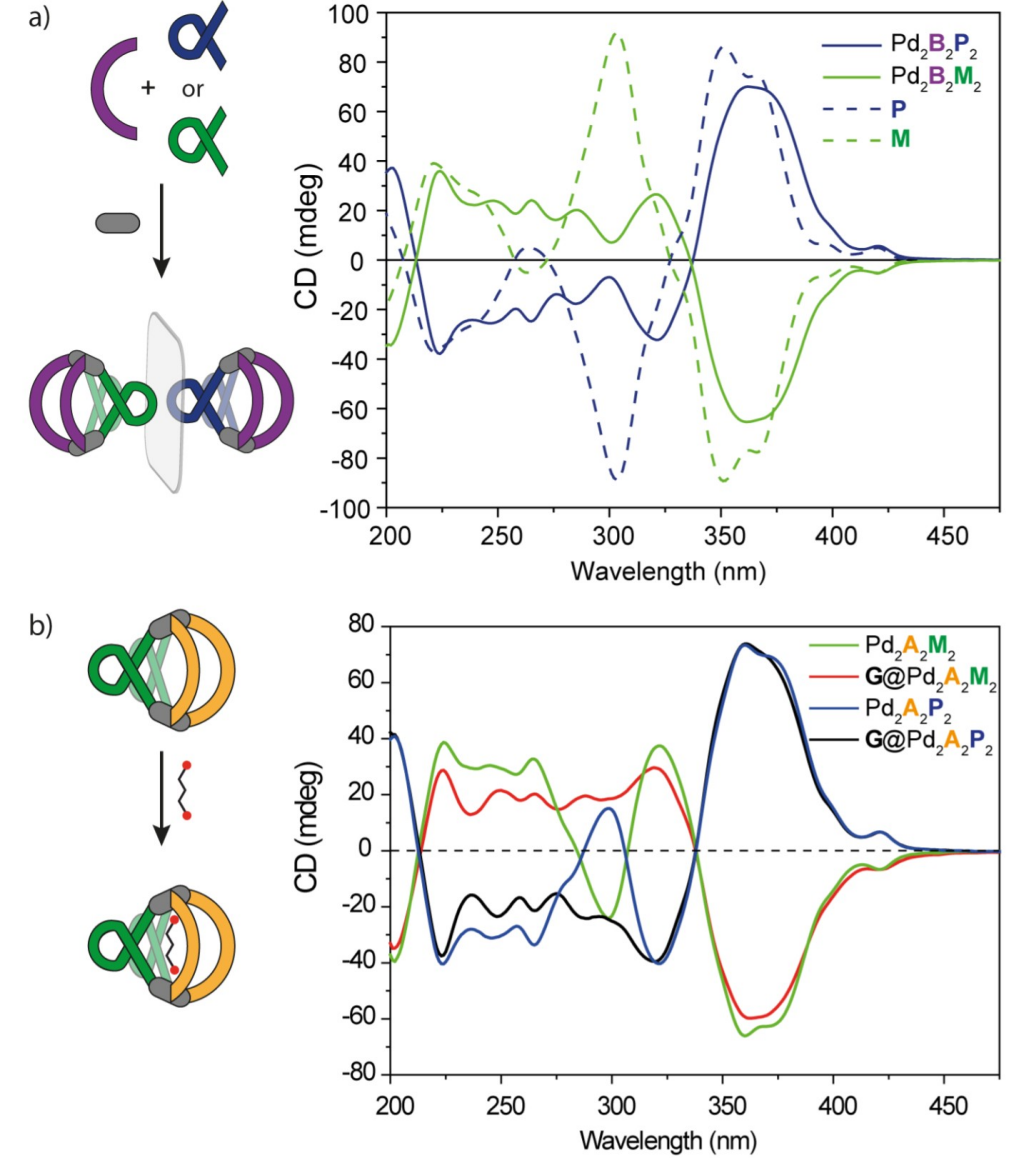
a) CD spectra of ligands **M**, **P** and heteroleptic cages Pd_2_ **B**_2_ **M**_2_ and Pd_2_ **B**_2_ **P**_2_; b) CD spectra of heteroleptic cages Pd_2_ **A**_2_ **M**_2_, Pd_2_ **A**_2_ **P**_2_, and host–guest complex **G**@Pd_2_ **A**_2_ **M**_2_ and **G**@Pd_2_ **A**_2_ **P**_2_.

The heteroleptic cages Pd_2_ **A**_2_(**M**/**P**)_2_ are characterized by a broad absorption band between 275 and 300 nm and a shoulder around 350 nm, that basically corresponds to the superposition of both ligands. Using the helicene based ligands as enantiomerically pure compound bestows the system with a chiroptical fingerprint. CD spectra in acetonitrile of both **M** and **P** ligands show strong bands at 350 and 300 nm, with a negative exciton couplet for the M enantiomer, and perfect mirror image for the P enantiomer (Figure 4a).^[32]^ CD spectra of the homoleptic cages Pd_2_ **M**_4_ and Pd_2_ **P**_4_ follow the same behaviour observed in the absorption spectra, with a bathochromic shift of the bands (Figure S22). We then studied the heteroleptic cages, to see if the chiral information imparted from the helicenes to the overall structure is transferred to the fluorenone‐based ligands. CD spectra of Pd_2_ **A**_2_(**M**/**P**)_2_ (Figure 4b) and Pd_2_ **B**_2_(**M**/**P**)_2_ (Figure 4a) heteroleptic cages show similar bands as the homoleptic analogues, with some differences mostly in the 275–300 nm region. Unfortunately, the big overlap between the fluorenone and the helicene ligands’ absorption bands prevent a clear answer whether it is possible to ascribe a CD contribution also to the non‐chiral ligands.

**Figure 5:**
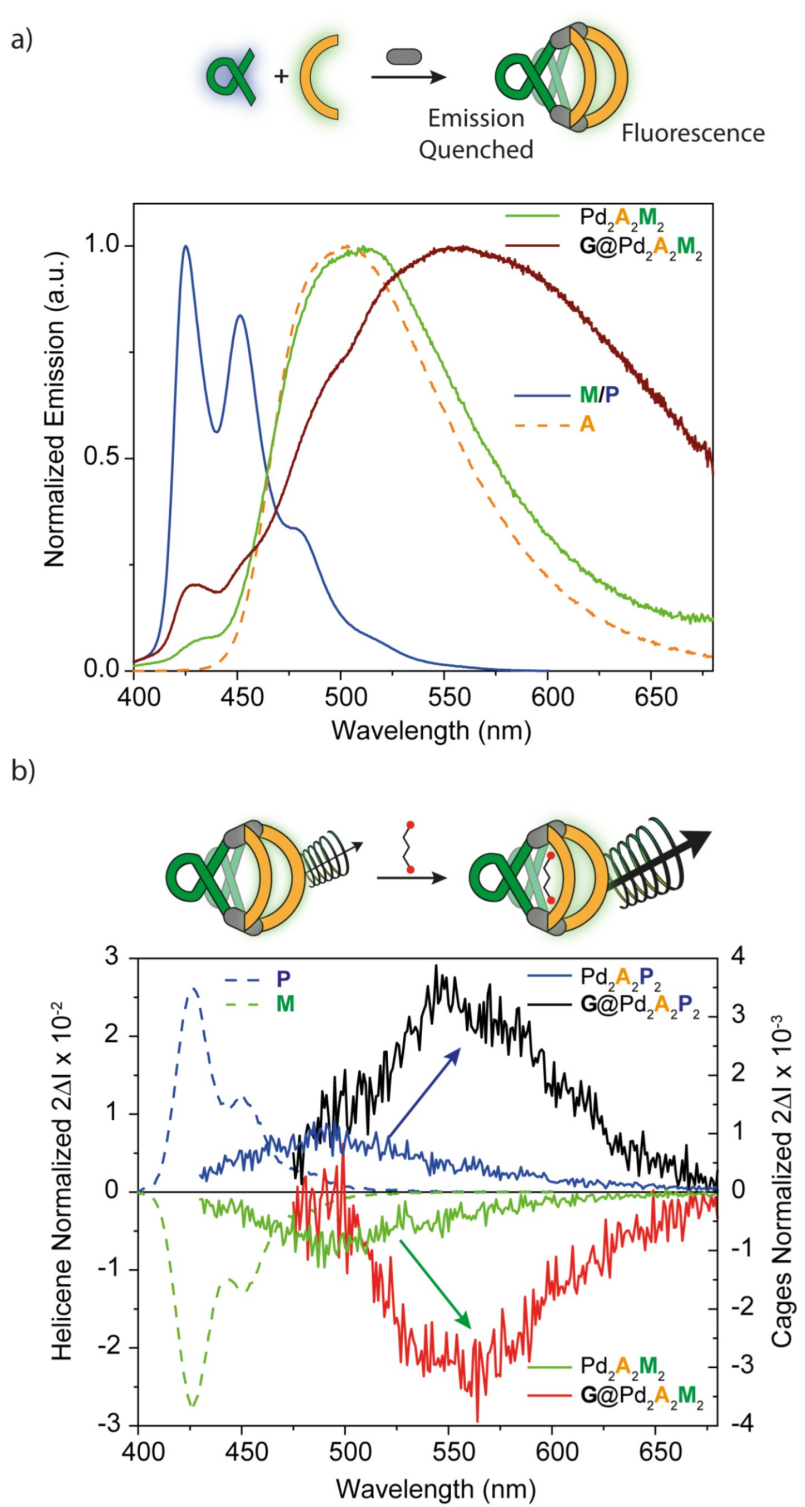
a) Emission spectra (CD_3_CN, *λ*_ex_=350 nm) of ligands **M**/**P** and **A**, heteroleptic cage Pd_2_ **A**_2_ **M**_2_ and host–guest complex **G**@Pd_2_ **A**_2_ **M**_2_; b) CPL spectra of ligands **M**, **P**, heteroleptic cages Pd_2_ **A**_2_ **M**_2_, Pd_2_ **A**_2_ **P**_2_, and host–guest complexes **G**@Pd_2_ **A**_2_ **M**_2_ and **G**@Pd_2_ **A**_2_ **P**_2_.

To overcome this problem, we then studied the emission properties of the system. Both helicene and fluorenone moieties have fluorescence properties, but emit in different spectral regions. Upon excitation at 350 nm, ligand **M**/**P** displays a typical structured blue emission with a maximum at 426 nm and additional vibronic pattern at 452, 480 and 520 nm, while ligand **A** exhibits a broad emission band around 500 nm (Figure 5a). In addition, helicene‐based compounds are well known systems for having remarkable CPL properties, that can be boosted when forming a push‐pull system, for instance embedding pyridine substituents, as recently shown by the group of Crassous and Favereau^[63]^ or by combining helicenes with phosphorescent Pt^II^ moieties, as introduced by Fuchter et al.^[64]^ Ligand **M**/**P**, bearing a *meta*‐pyridine donor group, shows a relatively strong CPL effect with |*g*_lum_|=2.8×10^−2^ (Figure 5b), perfectly in line with the reported analogue bearing a *para*‐pyridine substituent.^[63]^ However, the fluorescence, and therefore the CPL properties of ligand **M**/**P** were completely quenched after coordination to Pd^II^ cations, an effect that is frequently observed for conjugated luminescent ligands coordinating to transition metal ions with a non‐d^10^ electron configuration.^[49]^ It is worth noting that only few examples of emissive Pd^II^‐based cages were reported so far,[^29^, ^49^] most of which possessing either Ru^II^‐based emitters,[^23^, ^28^, ^65^] or embedding the luminophores as external pendant.[^26^, ^27^] However, we previously reported a system where the emission properties of a fluorenone‐based ligand are maintained in both homo‐ and heteroleptic Pd^II^‐based cages.^[49]^ Indeed, also here this is the case (emission quantum yields given in Table S1). The homoleptic cage Pd_2_ **A**_4_ and the heteroleptic cage Pd_2_ **A**_2_(**M**/**P**)_2_ show a broad emission band around 510 nm, slightly red‐shifted compared to the free ligand (Figure 5a). Likewise, Pd_2_ **B**_2_(**M**/**P)**_2_ exhibit an emission at 520 nm (Figure S24). Interestingly, all the heteroleptic cages exhibit a CPL effect, matching the fluorescence spectra and therefore originating from the non‐chiral fluorenone ligands **A** or **B** (Figure 5b). In order for this to happen, the chiral information from **M** or **P** must be transferred to the overall supramolecular system, inducing a twist in the Pd^2+^ coordination environments, and therefore to the fluorenone‐based ligands as well. The CPL intensity is decreased compared to what is achieved by the helicene ligand alone, with |*g*_lum_| values of 0.9×10^−3^ for Pd_2_ **A**_2_(**M**/**P**)_2_ and 0.4×10^−3^ for Pd_2_ **B**_2_(**M**/**P**)_2_. However, the chiroptical properties derive from the synergistic interaction of the two different ligands, mediated by the Pd^II^ metal centres, and thus mark the first multifunctional heteroleptic cage assembly showing a CPL emission. Interestingly, the two fluorenone ligands differ only by the nature of the linker, being an alkyne for **A** and a 1,4‐phenylene group for **B**. This variation results in a difference of the |*g*_lum_| values in the two heteroleptic cages. Trying to rationalize this, we compared the DFT models of the two heteroleptic cages. As has been shown previously in theory^[66]^ and experiment,^[32]^ a variation of helicene's helical pitch can affect its CD effect. Here, we envision that the length differences of counterparts **A** and **B** modulate the helical pitch of the helicene ligand, resulting in a different degree of chirality transfer. The helical pitch in Pd_2_ **A**_2_ **M**_2_ is smaller than that in Pd_2_ **B**_2_ **M**_2_ (3.82 Å vs. 3.97 Å, Figure 3b, c), presumably leading to the observed differences in the CPL induction. Alternatively, also the slightly different electronic situations of alkyne‐linked **A** as compared to phenylene‐linked **B** may play a role.

Having shown that our heteroleptic cages are able to bind a guest molecule, we then investigated the effect of the molecular recognition process on the chiroptical properties. Addition of **G** to both heteroleptic cages leads to a modulation of the CD spectra, especially in the UV region below 325 nm, with main changes involving once more the band around 275–300 nm (Figure 4b, S23). As this area features the absorption contribution of both ligands, the modulation of the overall CD spectrum is difficult to interpret. On the contrary, CPL analysis provides a simpler output to monitor the host–guest interaction. Notably, formation of the host–guest systems **G**@Pd_2_ **A**_2_(**M**/**P**)_2_ and **G**@Pd_2_ **B**_2_(**M**/**P**)_2_ results in a pronounced bathochromic shift (50 and 40 nm, respectively. See Supporting Information for TD‐DFT data supporting an electrostatic modulation of the emissive ligand's frontier orbital gap by the bound guest) of the emission, together with an amplification of the CPL effect (Figure 5b, S24). In both cases we observed a 4‐fold increase of |*g*_lum_|, achieving values of 3.5×10^−3^ for **G**@Pd_2_ **A**_2_(**M**/**P**)_2_ and 1.5×10^−3^ for **G**@Pd_2_ **B**_2_(**M**/**P**)_2_. DFT analysis, as well as ion mobility data, shows how accommodation of the guest inside the cavity, results in a shrinkage of the cage, with a reduction of the Pd⋅⋅⋅Pd distances. A narrowing effect on the CCS signal as observed when comparing insets in Figure 2b and c may further point to a loss of overall structural flexibility upon guest encapsulation. We postulate that this structural variation contributes to the observed shift and intensity increase of the CPL signal, however, the role of an electronic contribution from the negatively charged guest should also be taken into account.

## Conclusion

In conclusion, we successfully applied the shape‐complementary assembly approach (SCA) to achieve integrative self‐sorting of a series of CPL‐active heteroleptic Pd_2_L_2_L′_2_ cages, for the first time. The overall properties derive from the synergy of the three building blocks, one ligand (**M**/**P**) carries the chiral information, the other ligand (**A** or **B**) brings emission properties, while coordination to Pd^II^ cations allows to create a discrete cavity. The spring‐like behaviour of the helicene ligands allows to control their helical pitch, adapting in size to match the second ligand and adjust to guest binding. The chiroptical consequences of combining the ligands into a self‐assembled cage and the guest influence were studied by CD spectroscopy, however, complicated by the overlap of the absorption bands of both ligands. On the other hand, CPL analysis allowed to unambiguously show the chirality transfer and guest modulation in the system by limiting the readout to only the emissive ligand, hence simplifying the chiroptical analysis. We demonstrated how a rather simple modification of the non‐chiral ligand can result in a change in CPL intensity, paving the way for new strategies to achieve modular tunability of chiroptical properties in self‐assembled compounds and materials. Furthermore, guest recognition was shown to shift and enhance the CPL output, showing the potential of such systems for molecular recognition and imaging applications based on a chiroptical readout.

## Conflict of interest

The authors declare no conflict of interest.

1

## Supporting information

As a service to our authors and readers, this journal provides supporting information supplied by the authors. Such materials are peer reviewed and may be re‐organized for online delivery, but are not copy‐edited or typeset. Technical support issues arising from supporting information (other than missing files) should be addressed to the authors.

Supporting Information

## Data Availability

The data that support the findings of this study are available from the corresponding author upon reasonable request.
