# Supplementary material for: Guest‐Modulated Circularly Polarized Luminescence by Ligand‐to‐Ligand Chirality Transfer in Heteroleptic PdII Coordination Cages
Source: Angew Chem Int Ed Engl. 2022 Jul 4;61(35):e202205725. doi: 10.1002/anie.202205725 (PMC9544203; doi:10.1002/anie.202205725)
Supplement: Supplementary file 1 — Supporting Information [file ANIE-61-0-s001.pdf]

Angewandte Chemie
Eine Zeitschrift der Gesellschaft Deutscher Chemiker

Supporting Information

Guest-Modulated Circularly Polarized Luminescence by Ligand-to-Ligand Chirality Transfer in Heteroleptic Pd^{II} Coordination Cages

*K. Wu, J. Tessarolo, A. Baksi, G. H. Clever**

Supporting Information

Table of Contents

1. Experimental section 2
1.1. Materials and measurements 2
1.2. Self-assembly and characterization of ligands and cages 2
1.2.1. Synthesis of 3,6-bis(4-(pyridin-3-yl)phenyl)-9H-fluoren-9-one 2
1.2.2. Self-assembly of homoleptic cage **$[Pd_2(M/P)_4](BF_4)_4$** in DMF-d_7 3
1.2.3. Self-assembly of heteroleptic cage **$[Pd_2A_2(M/P)_2](BF_4)_4$** in CD_3CN 5
1.2.4. Self-assembly of heteroleptic cage **$[Pd_2B_2(M/P)_2](BF_4)_4$** in CD_3CN 8
2. Host-Guest chemistry study 11
3. Photophysical properties 14
4. Ion Mobility Mass Spectrometry 16
5. Computational studies 18
6. X-ray Crystallography 20
6.1. Crystal structure of $[Pd_2M_4](BF_4)_4$ 21
6.1.1. Specific refinement details. 21
7. References 21

1. Experimental section

1.1. Materials and measurements

Unless otherwise stated, all chemicals were obtained from commercial sources and used as received.

Ligands **A**,[1] enantiopure helicene **M**/**P**[2] and homoleptic cage[1] $[Pd_2\mathbf{A}_4]^{4+}$ were prepared according to literature procedures. Gel permeation chromatography (GPC) purification of ligands was performed on a JAI 9210-II NEXT GPC System with a JAIGEL HH-2/HH-1 column combination running with $CHCl_3$ (HPLC grade). High resolution Electrospray Ionization (HR-ESI) mass spectra and trapped ion mobility data were recorded on Bruker ESI-timsTOF and (electrospray ionization-trapped ion mobility-time of flight) Compact mass spectrometers. All samples were diluted with spectroscopic grade CH_3CN (1:10) prior to measurement. NMR experiments were measured on Bruker AVANCE III and NEO (500, 600 or 700 MHz) spectrometers. Chemical shifts for ^{1}H and ^{13}C are reported in ppm with residual solvent as reference: Acetonitrile (1.94 ppm for ^{1}H, 1.32 ppm for ^{13}C), DMSO (2.50 ppm for ^{1}H, 39.52 ppm for ^{13}C), DMF (2.75 ppm for ^{1}H, 29.76 ppm for ^{13}C). Abbreviations for signal multiplicity of ^{1}H NMR spectra are shown as following: s: singlet, d: doublet, t: triplet, dd: doublet of doublets; dt: doublet of triplets; m: multiplet, br: broad. UV-vis spectra were recorded on a DAD HP-8453 UV-Vis spectrometer. Circular dichroism spectra were recorded in CD_3CN with an Applied Photophysics Chirascan qCD Spectrometer with a temperature-controlled cuvette holder. The spectra were background-corrected and smoothed with a window size of 5. Emission measurements were performed on a JASCO FP-8300, quantum yield determination have been performed on a JASCO ILF-835 integrating sphere as accessory of the JASCO FP-8300, circularly polarized luminescence measurements were performed using a JASCO CPL-300 spectrophotometer, both equipped with a (150 W) Xe lamp as light source. CPL spectra of ligand **M** and **P** were recorded with excitation bandwidth of 25 nm and emission bandwidth of 15 nm and averaged over 10 spectra. CPL of heteroleptic cages and host guest complexes were recorded with excitation and emission bandwidth of 30 nm and averaged over 10 spectra.

1.2. Self-assembly and characterization of ligands and cages

1.2.1. Synthesis of 3,6-bis(4-(pyridin-3-yl)phenyl)-9H-fluoren-9-one

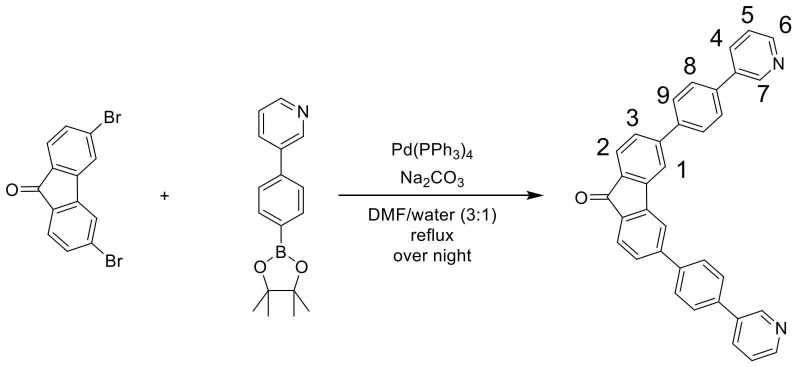

B

Scheme 1. Synthetic route for ligand **B**.

3,6-dibromo-9*H*-fluoren-9-one (250 mg, 0.74 mmol, 1 equiv.), 3-(4-(4,4,5,5-tetramethyl-1,3,2-dioxaborolan-2-yl)phenyl)pyridine (500 mg, 1.78 mmol, 2.4 equiv.), Na_2CO_3 (235 mg, 2.22 mmol, 3 equiv.) and $Pd(PPh_3)_4$ (25.6 mg, 0.022 mmol, 0.03 equiv.) were suspended in a mixture of DMF/H_2O (3:1, 12 ml). The mixture was degassed three times using the *Freeze-Pump-Thaw*-method, heated to reflux and stirred overnight. After cooling down to room temperature, the reaction mixture was extracted with chloroform. The organic phase was washed consecutively with water and then brine. It was dried over $MgSO_4$, filtered and the solvent was removed under reduced pressure. The crude product was purified by column chromatography (SiO_2; $CHCl_3$/methanol; 0 to 10 %) yielding a yellow powder that was further purified by GPC. The yield of the final product was 87 mg (0.179 mmol, 24%).

^{1}H NMR (700 MHz, 298 K, DMSO-*d*$_6$) δ 9.03 (d, ^4J = 2.0 Hz, 2H, H7), 8.63 (dd, ^3J = 4.7, ^4J = 1.05 Hz, 2H, H6), 8.48 (s, 2H, H1), 8.24 (d, ^3J = 7.9 Hz, 2H, H4), 8.03 (d, ^3J = 8.3 Hz, 4H, H9), 7.95 (d, ^3J = 8.3 Hz, 4H, H8), 7.82 (d, ^3J = 7.7, ^4J = 1.05 Hz, 2H, H3), 7.75 (d, ^3J = 7.7 Hz, 2H, H2), 7.57 (d, ^3J = 7.7, 4.7 Hz, 2H, H5) ppm.

^{13}C NMR (176 MHz, 298 K, DMSO-*d*$_6$) δ 192.16 (C_{XVI}), 148.42 (C_{II}), 147.35 (C_{I}), 146.06 (C_{X}), 144.65 (C_{XIV}), 138.66 (C_{IX}), 137.15 (C_{VI}), 134.94 (C_{V}), 134.57 (C_{IV}), 132.93 (C_{XIII}), 127.78 (C_{VIII}), 127.67 (C_{XI}), 127.53 (C_{VII}), 124.46 (C_{XII}), 124.10 (C_{III}), 119.90 (C_{XV}) ppm.

HR ESI-MS: measured for $[C_{35}H_{22}N_2O+H]^+$: 487.1784, calculated: 487.1805.

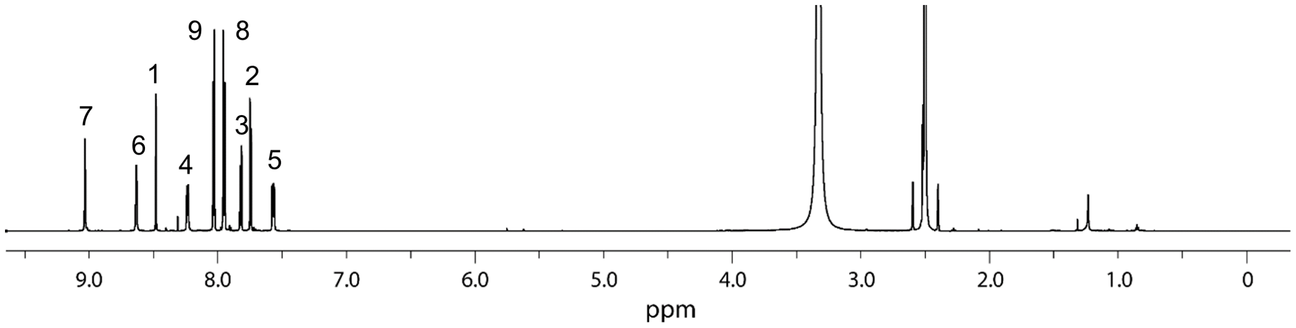

Figure S1. ^{1}H NMR spectrum (700 MHz, 298 K, DMSO-d_6) of ligand **B**.

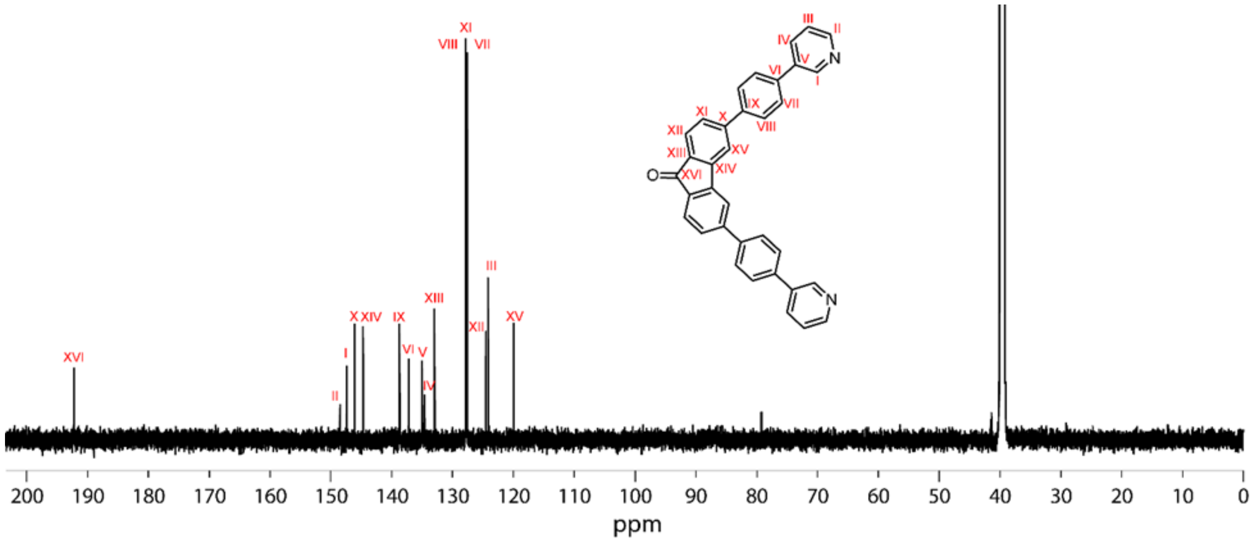

Figure S2. ^{13}C NMR spectrum (176 MHz, 298 K, DMSO-d_6) of **B**.

1.2.2. Self-assembly of homoleptic cage ***[Pd$_2$(M/P)$_4$](BF$_4$)$_4$*** *in DMF-d_7*

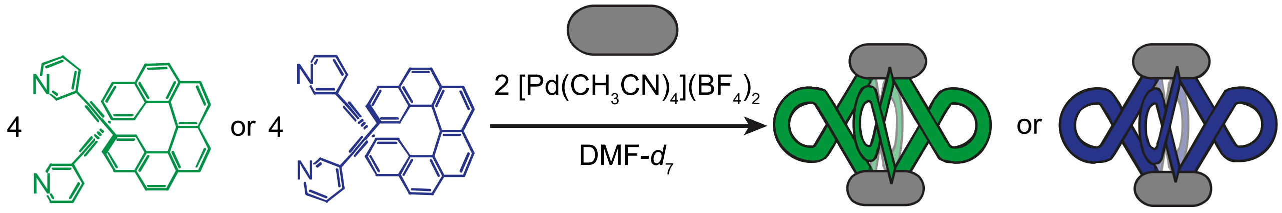

M **P** Pd$_2$**M**$_4$ Pd$_2$**P**$_4$

The homoleptic cage was prepared with a similar procedure as reported but with DMF-d_7 as solvent instead. To a solution of **M/P** (0.47 mg, 0.886 µmol) in 418 µL DMF-d_7 was added a stock solution of $[Pd(CH_3CN)_4](BF_4)_2$ (25 µL, 20 mM/DMF-d_7, 0.5 µmol). The mixture was heated at 80 °C overnight to give a 0.5 mM solution of cage.

^{1}H NMR (500 MHz, 298K, DMF-d_7) δ 9.45 (s, 8H), 9.31 (d, J = 5.7 Hz, 8H), 8.39 (d, J = 8.3 Hz, 8H), 8.32 (d, J = 8.3 Hz, 8H), 8.25 (d, J = 8.6 Hz, 8H), 8.22 (dt, J = 8.1, 1.6 Hz, 8H), 8.17 (d, J = 8.3 Hz, 8H), 8.12 (d, J = 8.6 Hz, 8H), 7.90 (s, 8H), 7.87 – 7.82 (m, 16H).

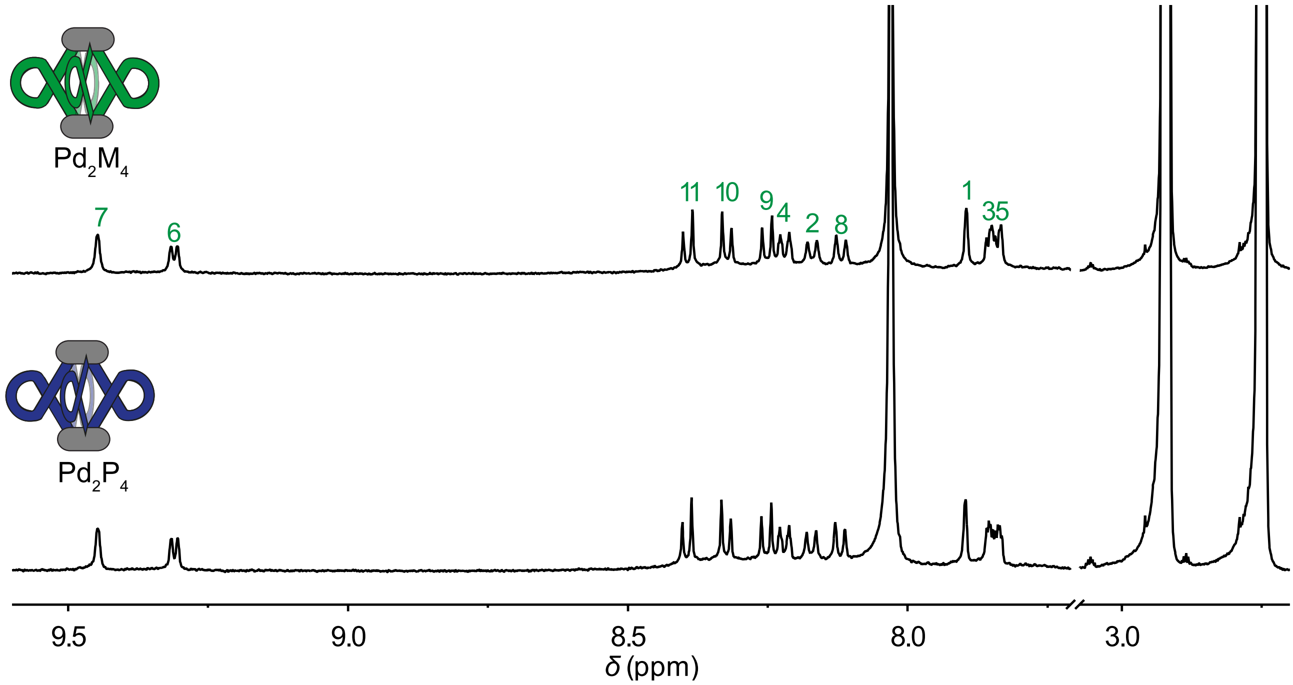

Figure S3. ^{1}H NMR spectrum (500 MHz, 298K, DMF-d_7) of homoleptic cages $[Pd_2(\mathbf{M/P})_4]^{4+}$.

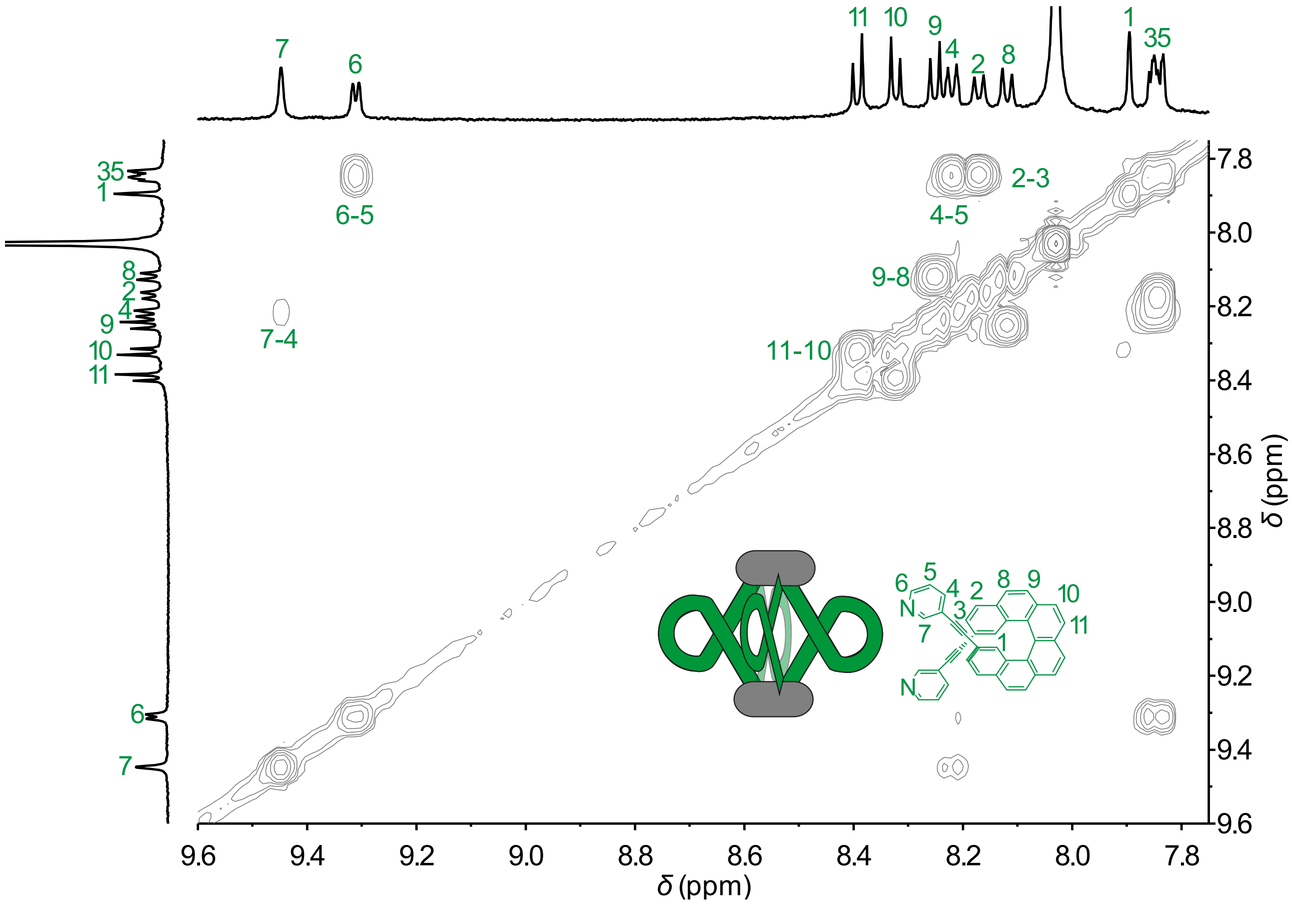

Figure S4. Partial ^{1}H–^{1}H COSY spectrum (500 MHz, 298K, DMF-d_7) of homoleptic cage $\mathbf{[Pd_2M_4]^{4+}}$.

*1.2.3. Self-assembly of heteroleptic cage **$[Pd_2A_2(M/P)_2](BF_4)_4$** in CD_3CN*

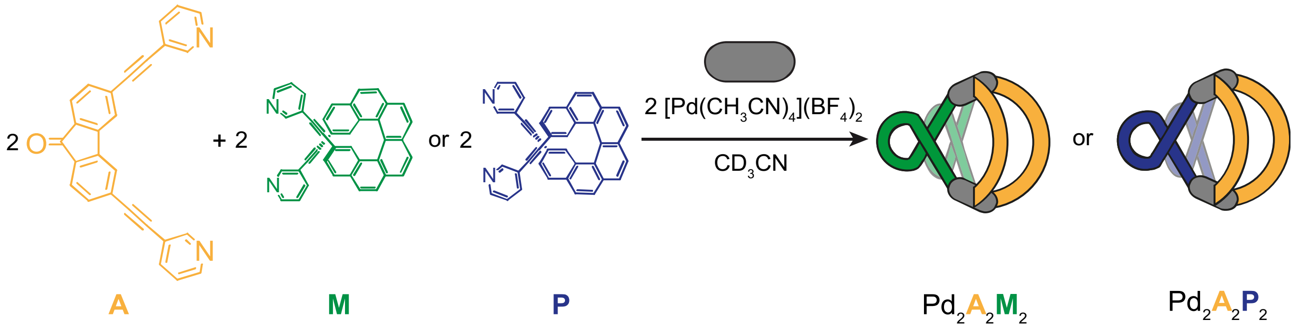

A suspension of **A** (0.092 mg, 0.24 µmol) and **M/P** (0.13 mg, 0.24 µmol) in 430 µL CD_3CN was combined. To this suspension, a stock solution of $[Pd(CH_3CN)_4](BF_4)_2$ (20 µL, 15 mM/CD_3CN, 0.3 µmol) was added. The mixture was heated at 80 °C for 8 h to give a 0.27 mM solution of heteroleptic cage.

^{1}H NMR (700 MHz, 298K, CD_3CN) δ 9.36 (d, J = 1.8 Hz, 2H), 9.27 (d, J = 1.8 Hz, 2H), 9.16 (d, J = 1.8 Hz, 2H), 9.02 (dd, J = 5.8, 1.4 Hz, 2H), 8.96 (dd, J = 5.0, 1.8 Hz, 4H), 8.90 (dd, J = 6.0, 1.4 Hz, 2H), 8.76 (dd, J = 6.1, 1.4 Hz, 2H), 8.70 – 8.66 (m, 2H), 8.31 (dt, J = 8.3, 1.6 Hz, 2H), 8.21 – 8.16 (m, 4H), 8.15 – 8.11 (m, 4H), 8.08 (dd, J = 8.4, 4.8 Hz, 4H), 8.01 (d, J = 8.8 Hz, 2H), 7.94 – 7.91 (m, 2H), 7.90 – 7.86 (m, 4H), 7.77 – 7.74 (m, 8H), 7.73 – 7.69 (m, 8H), 7.68 – 7.64 (m, 4H), 7.61 – 7.57 (m, 6H), 7.57 – 7.54 (m, 2H), 7.48 – 7.45 (m, 2H).

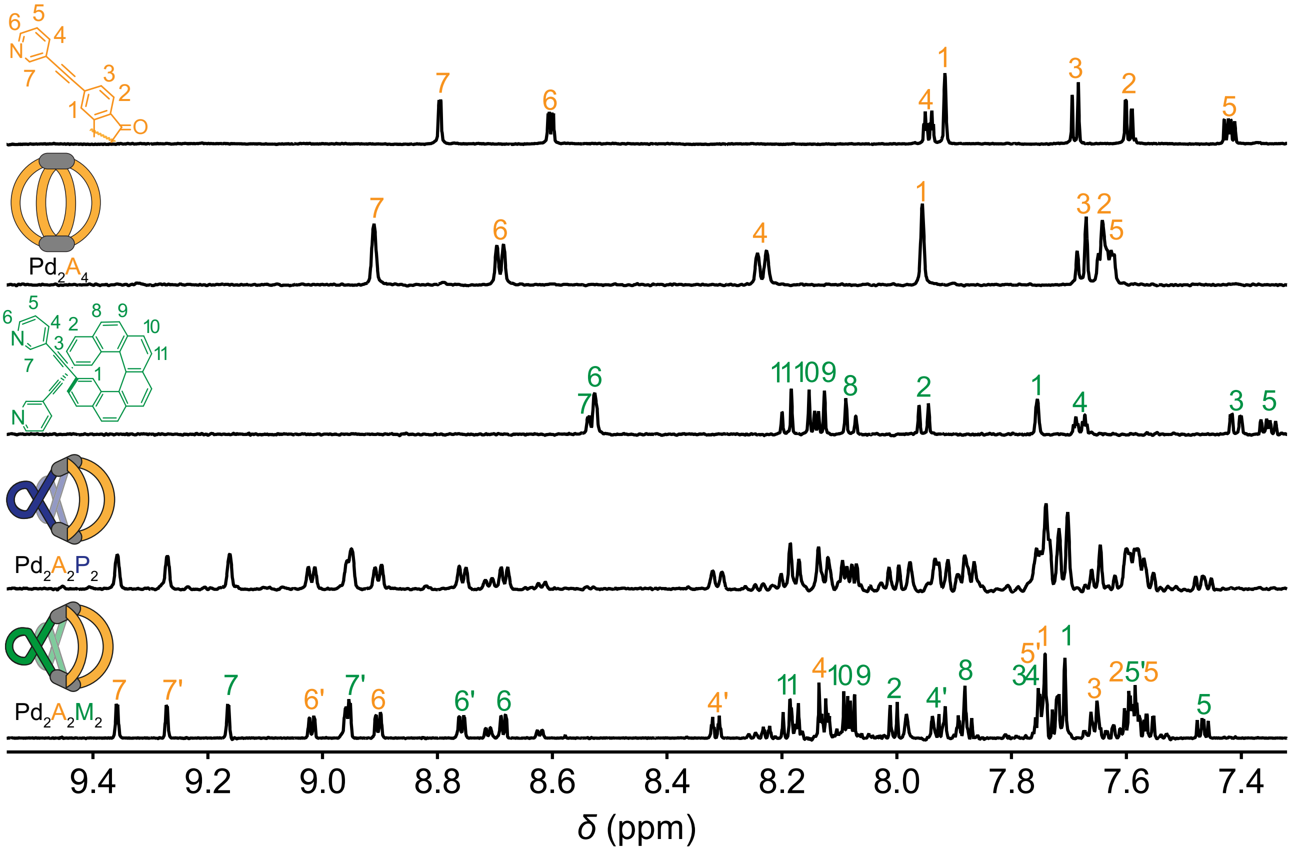

Figure S5. ^{1}H NMR spectra comparison (700 MHz, 298K, CD_3CN) of heteroleptic cages and ligands.

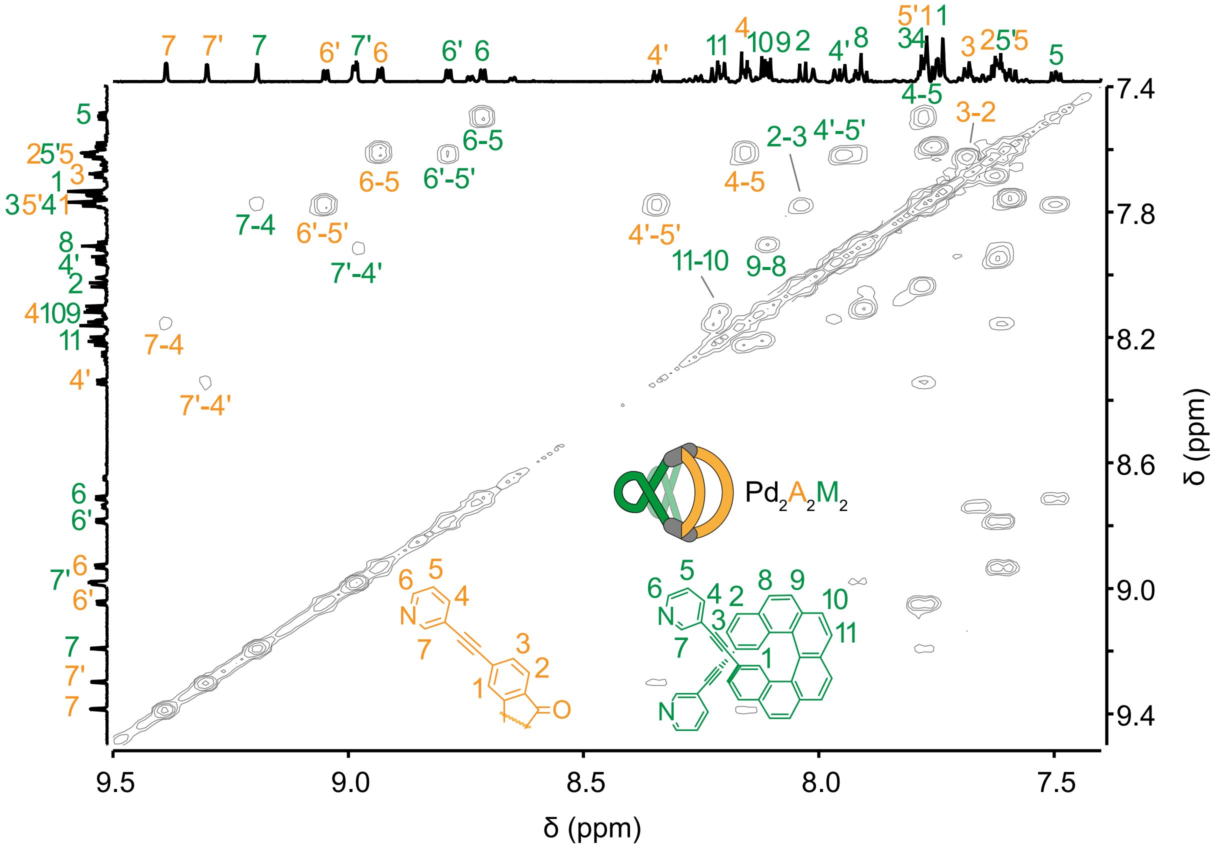

Figure S6. Partial ^{1}H–^{1}H COSY spectrum (500 MHz, 298K, CD_3CN) of heteroleptic cage $\mathbf{[Pd_2A_2M_2]^{4+}}$.

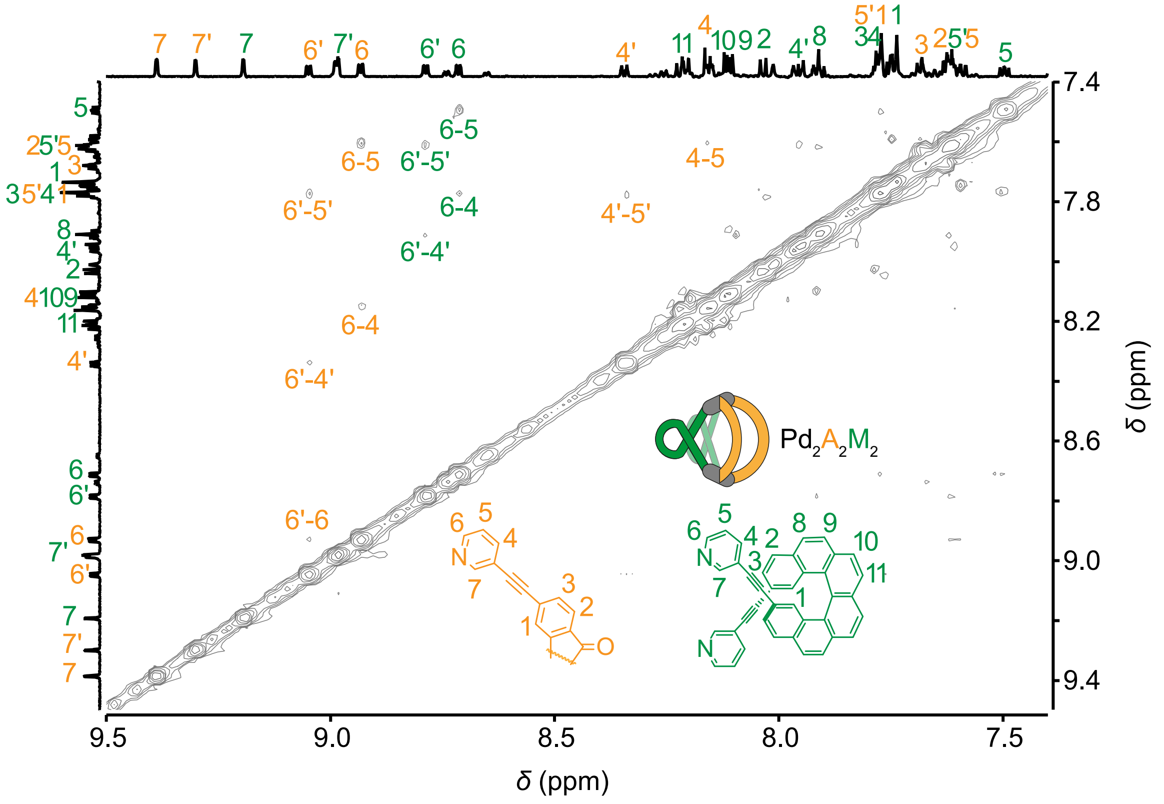

Figure S7. Partial ^{1}H–^{1}H NOESY spectrum (500 MHz, 298K, CD_3CN) of heteroleptic cage $\mathbf{[Pd_2A_2M_2]^{4+}}$.

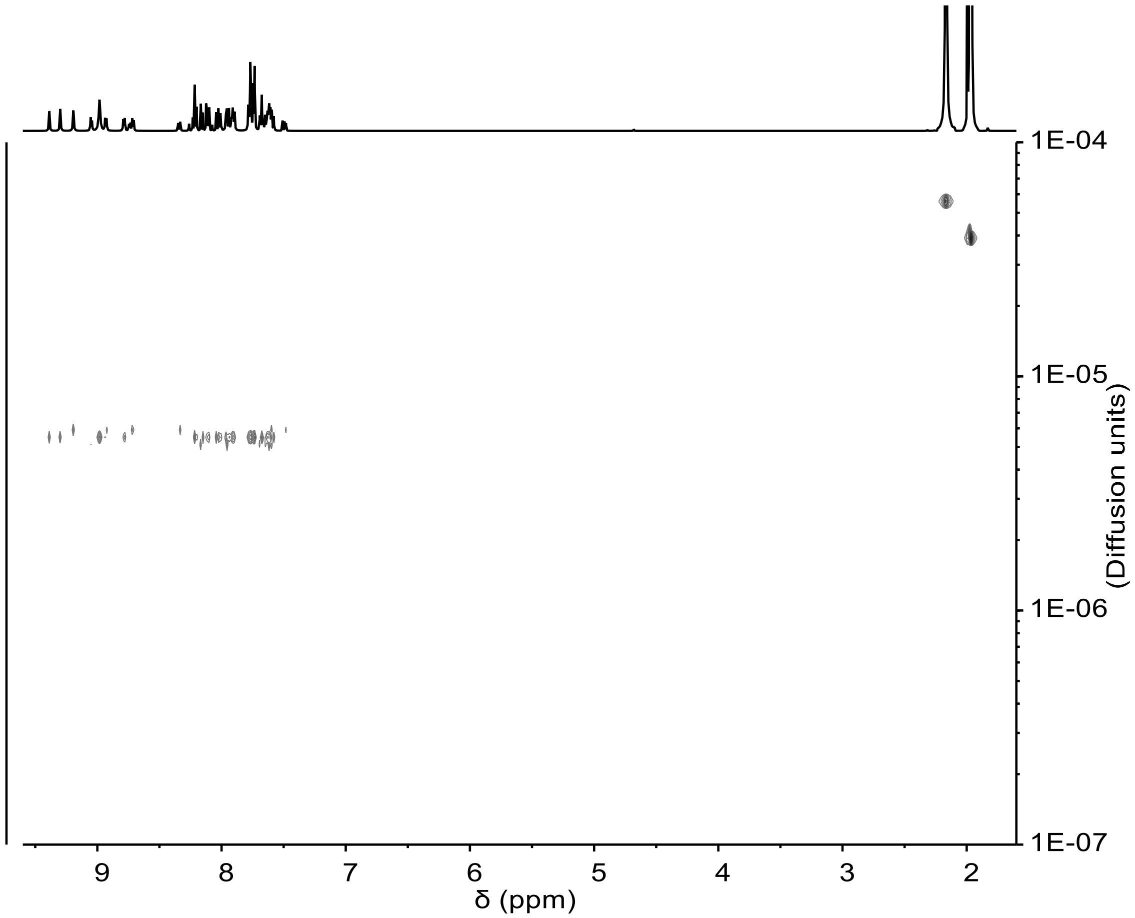

Figure S8. ^{1}H DOSY spectrum (500 MHz, 298K, CD_3CN) of heteroleptic cage **$[Pd_2A_2M_2]^{4+}$**. Diffusion coefficient: D = 5.85 x 10^{-10} m^2s^{-1}, r = 11.2 Å.

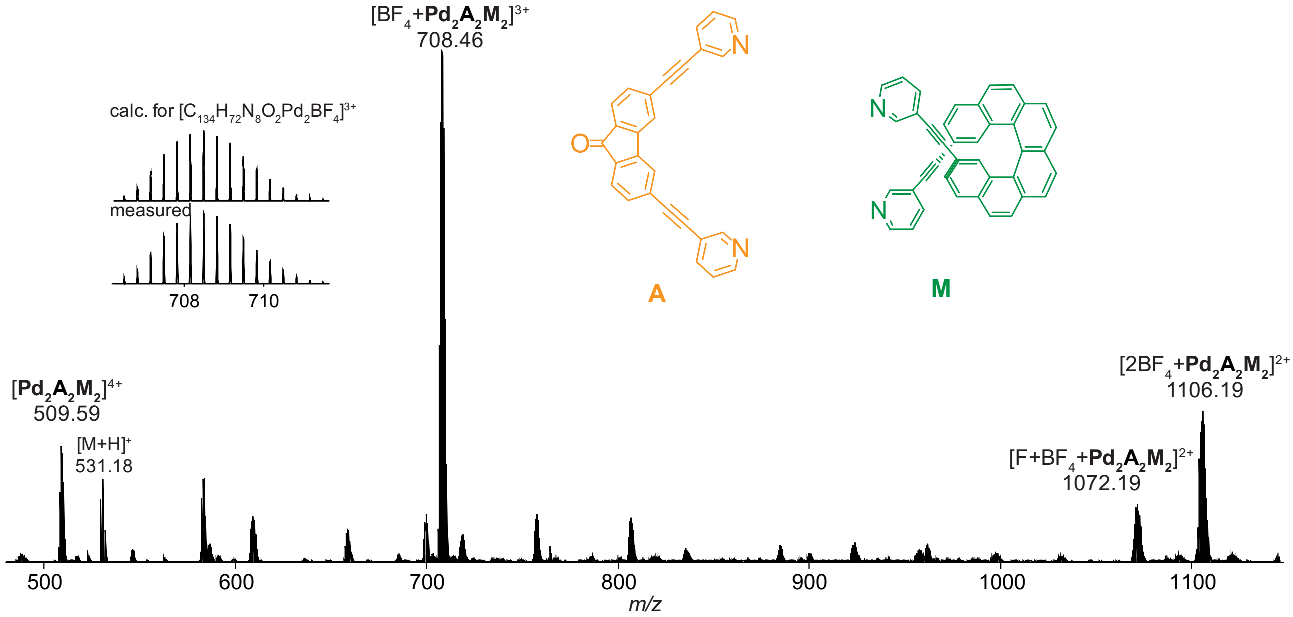

Figure S9. HR-ESI mass spectrum of heteroleptic cage **$[Pd_2A_2M_2]^{4+}$**.

1.2.4. Self-assembly of heteroleptic cage ***[Pd₂B₂(M/P)₂](BF₄)₄*** *in CD₃CN*

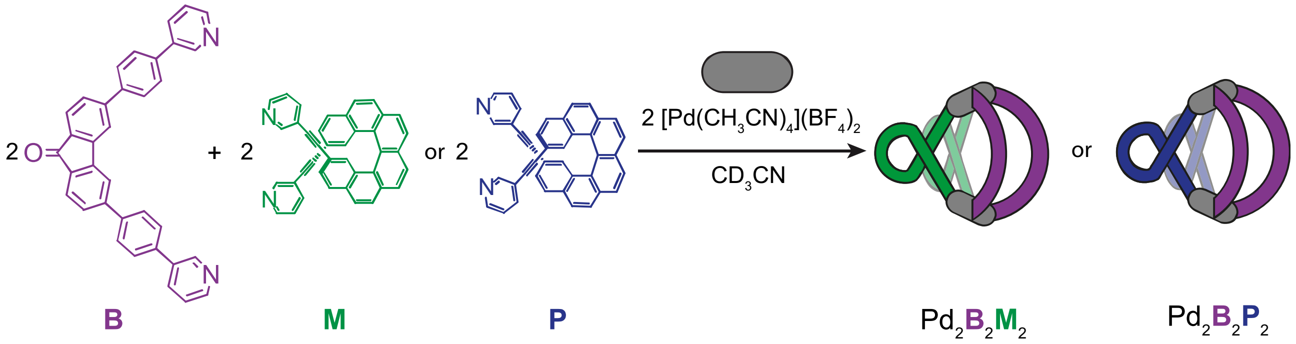

A suspension of **B** (0.12 mg, 0.24 μmol) and **M/P** (0.13 mg, 0.24 μmol) in 430 μL CD_3CN was combined. To this suspension, a stock solution of $[Pd(CH_3CN)_4](BF_4)_2$ (20 μL, 15 mM/CD_3CN, 0.3 μmol) was added. The mixture was heated at 80 °C for 8 h to give a 0.27 mM solution of heteroleptic cage.

^{1}H NMR (600 MHz, 298K, CD_3CN) δ 9.66 (d, J = 2.0 Hz, 2H), 9.48 (d, J = 1.8 Hz, 2H), 9.46 (d, J = 2.1 Hz, 2H), 8.99 (ddd, J = 7.7, 5.7, 1.2 Hz, 4H), 8.91 – 8.88 (m, 2H), 8.86 (d, J = 1.9 Hz, 2H), 8.73 (dd, J = 5.8, 1.3 Hz, 2H), 8.55 – 8.50 (m, 2H), 8.27 (d, J = 1.5 Hz, 2H), 8.27 – 8.23 (m, 2H), 8.18 (d, J = 1.6 Hz, 6H), 8.16 (s, 2H), 8.12 (dd, J = 10.3, 1.9 Hz, 4H), 8.09 – 8.06 (m,2H), 8.02 (d, J = 8.3 Hz, 2H), 7.94 (d, J = 8.8 Hz, 2H), 7.91 – 7.85 (m, 12H), 7.84 – 7.78 (m, 6H), 7.77 – 7.72 (m, 6H), 7.66 (t, J = 1.1 Hz, 4H), 7.65 – 7.60 (m, 2H), 7.57 – 7.54 (m, 2H), 7.52 – 7.45 (m, 4H), 7.35 (d, J = 8.6 Hz, 2H).

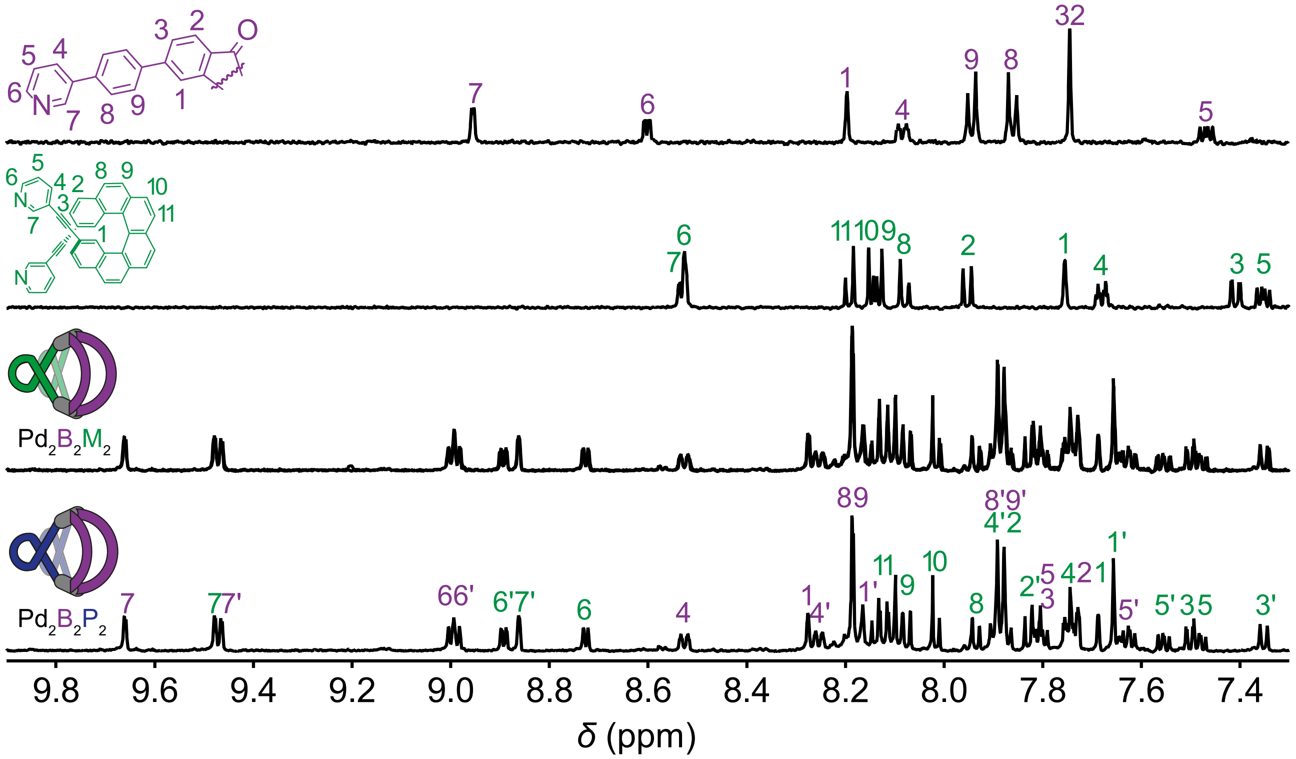

Figure S10. ^{1}H NMR spectra comparison (600 MHz, 298K, CD_3CN) of heteroleptic cages and ligands.

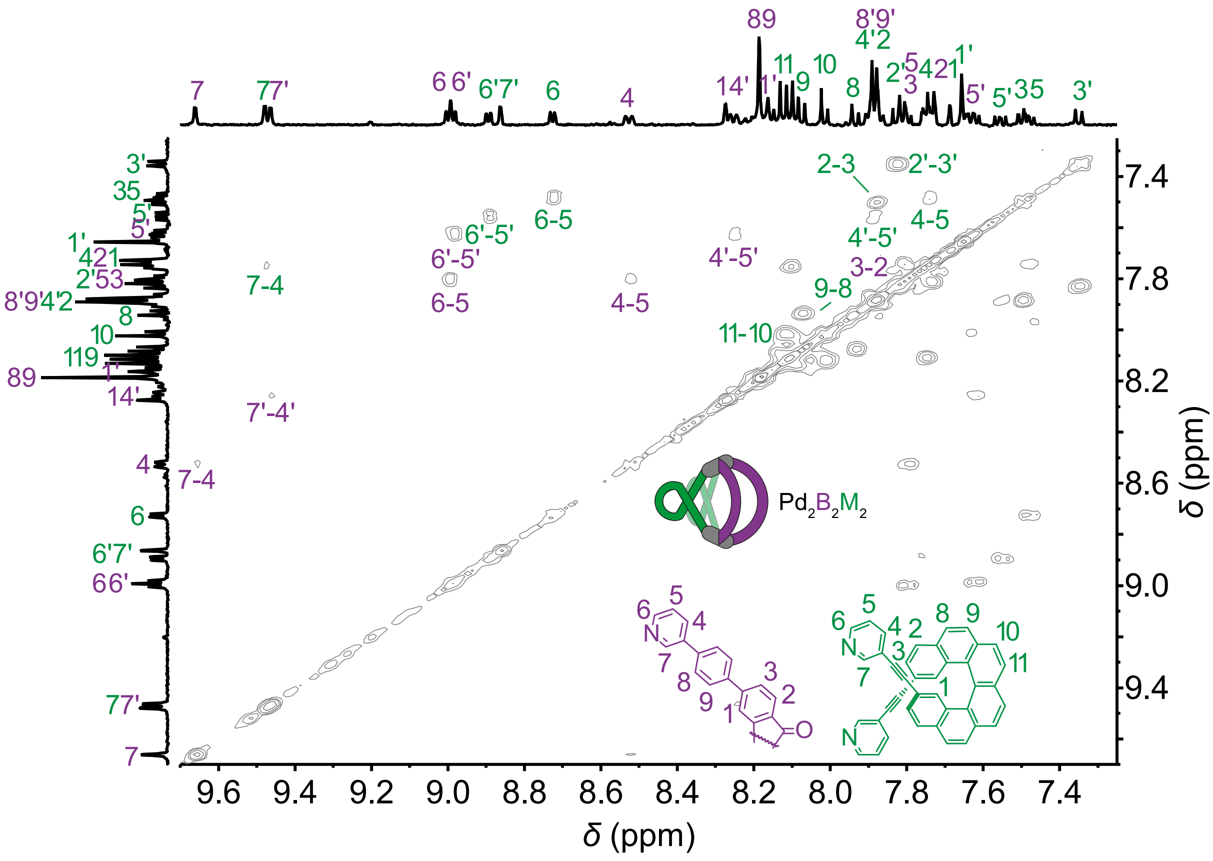

Figure S11. Partial ^1H–^1H COSY spectrum (500 MHz, 298K, CD_3CN) of heteroleptic cage [$\mathbf{Pd_2B_2M_2}$]$^{4+}$.

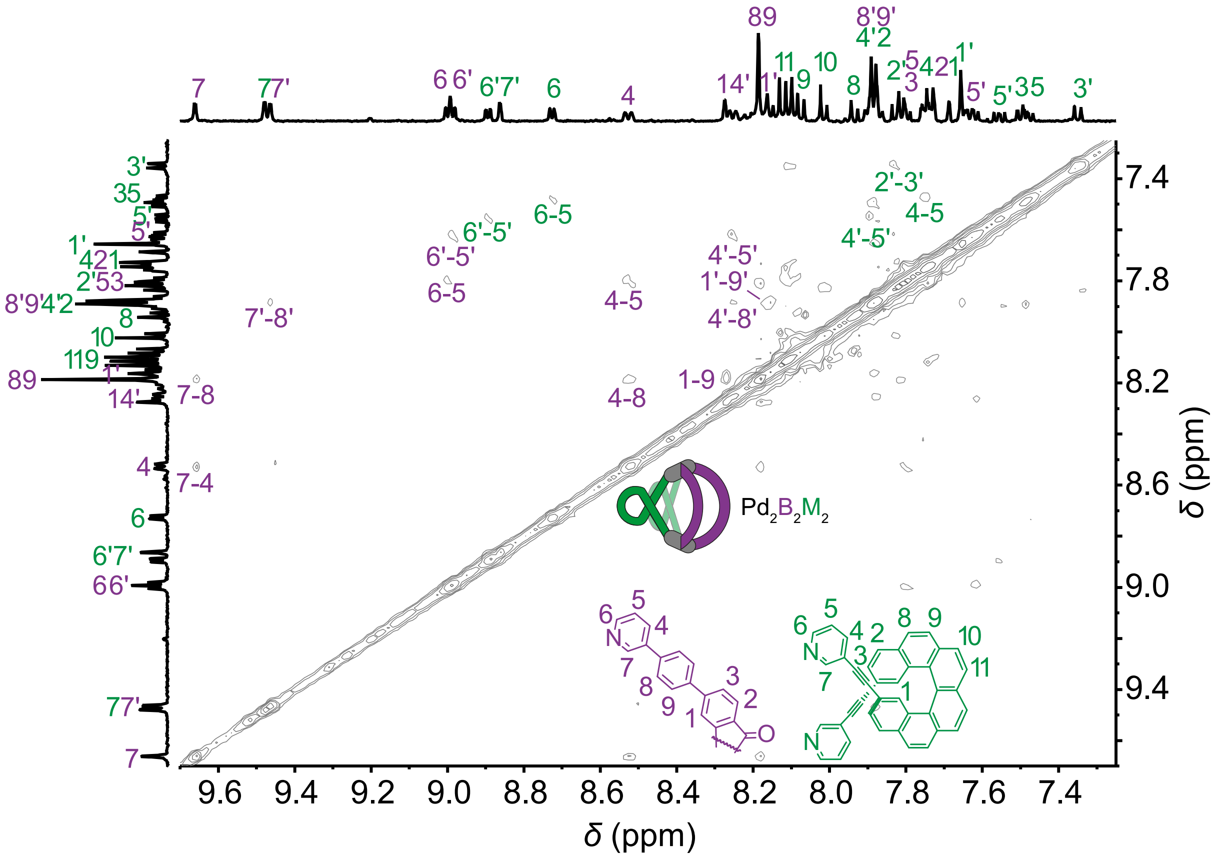

Figure S12. Partial ^1H–^1H ROESY spectrum (500 MHz, 298K, CD_3CN) of heteroleptic cage [$\mathbf{Pd_2B_2M_2}$]$^{4+}$.

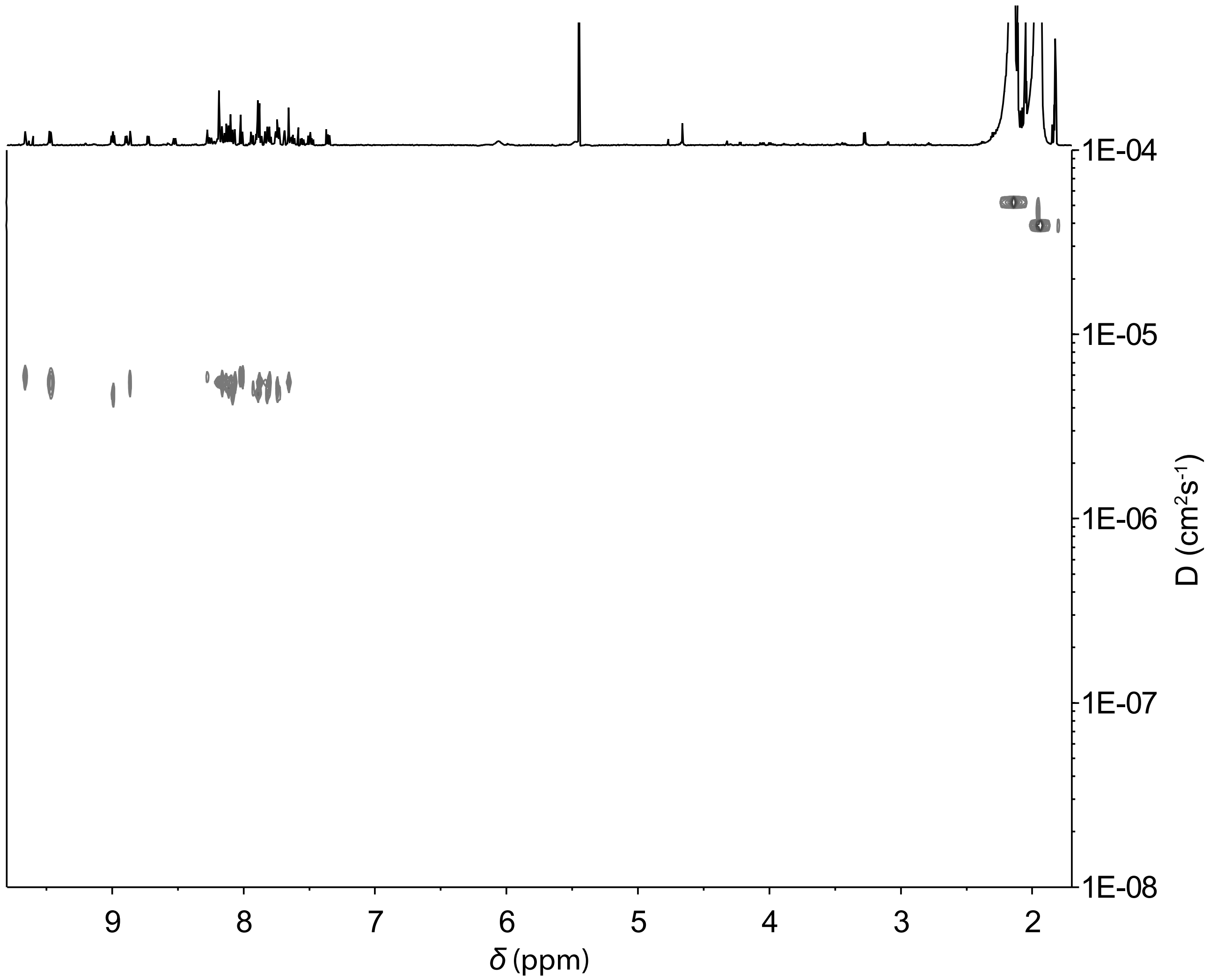

Figure S13. ^{1}H DOSY spectrum (500 MHz, 298K, CD_3CN) of heteroleptic cage $[\mathbf{Pd_2B_2M_2}]^{4+}$. Diffusion coefficient: D = 5.20 x 10^{-10} m^2s^{-1}, r = 12.6 Å.

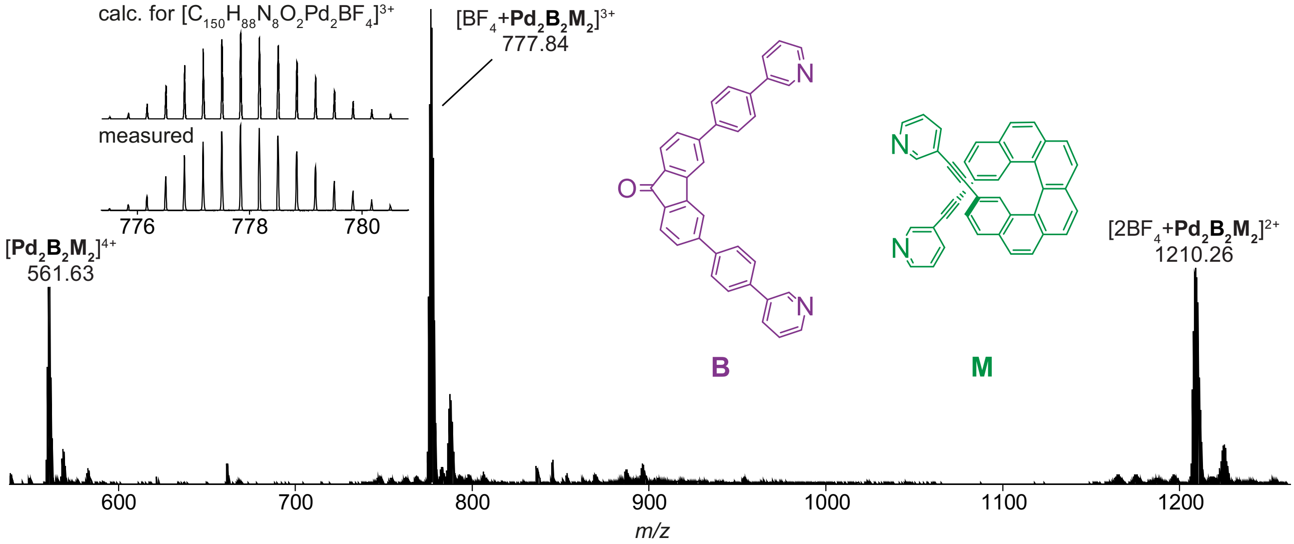

Figure S14. HR-ESI mass spectrum of heteroleptic cage $[\mathbf{Pd_2B_2M_2}]^{4+}$.

2. Host-Guest chemistry study

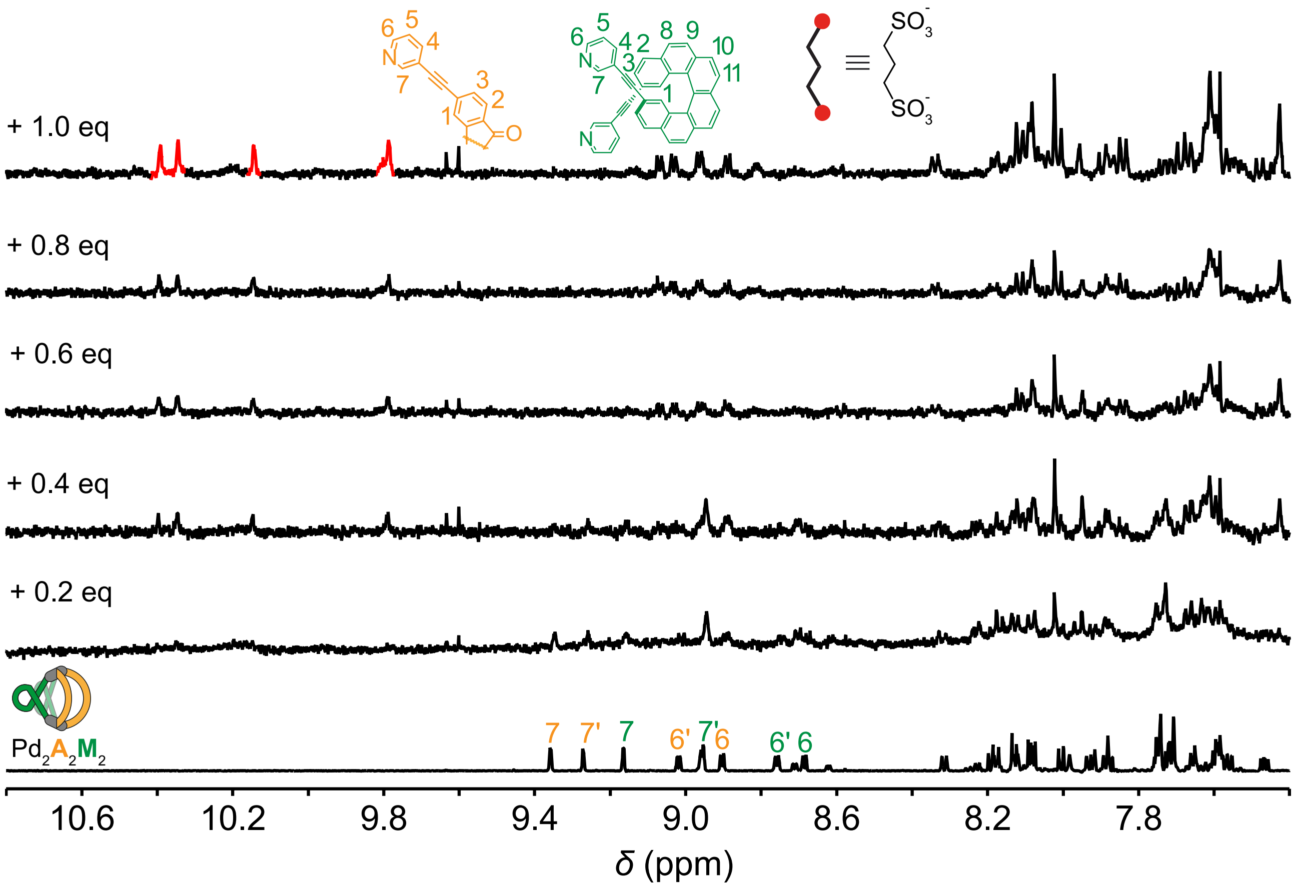

Figure S15. ^{1}H NMR titration (500 MHz, 298K, CD_3CN) of heteroleptic cage **$[Pd_2A_2M_2]^{4+}$** with bissulfonate guest in CD_3CN. The inward pointing protons 7 in the host-guest complex that show largest chemical shift are highlighted with red colour. The concurrent existence of both empty cage and host-guest complex indicates a slow exchange process for guest binding.

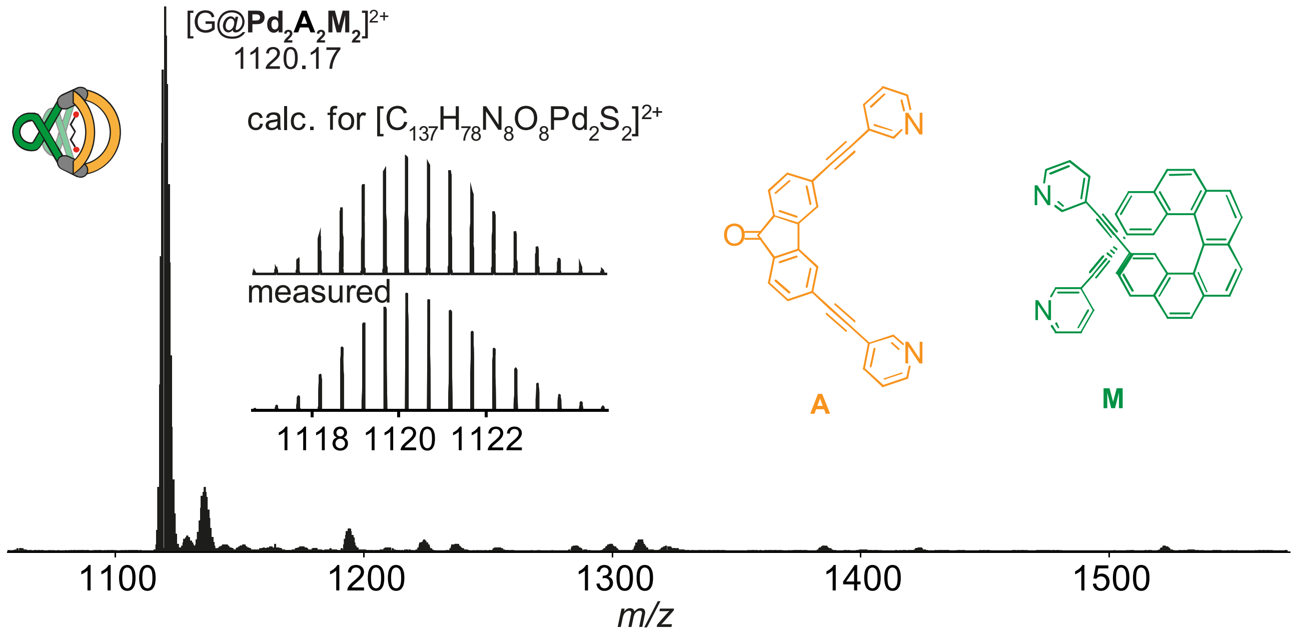

Figure S16. HR-ESI mass spectrum of heteroleptic cage **$[G@Pd_2A_2M_2]^{2+}$** (with 1 eq. of **G**).

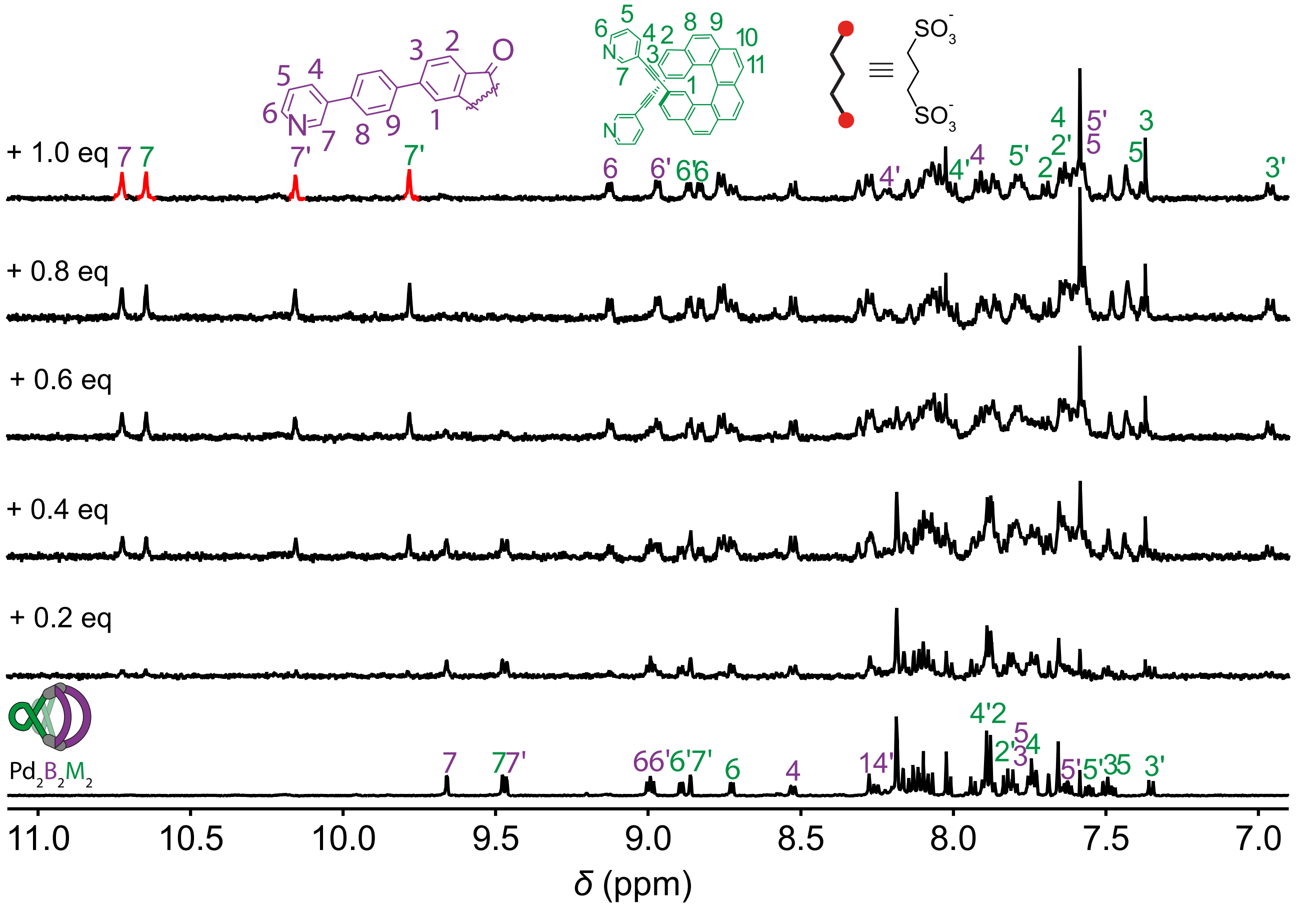

Figure S17. ^{1}H NMR titration (500 MHz, 298K, CD_3CN) of heteroleptic cage **$[Pd_2B_2M_2]^{4+}$** with bissulfonate guest in CD_3CN. The inward pointing protons 7 in the host-guest complex that show largest chemical shift are highlighted with red colour. The concurrent existence of both empty cage and host-guest complex indicates a slow exchange process for guest binding.

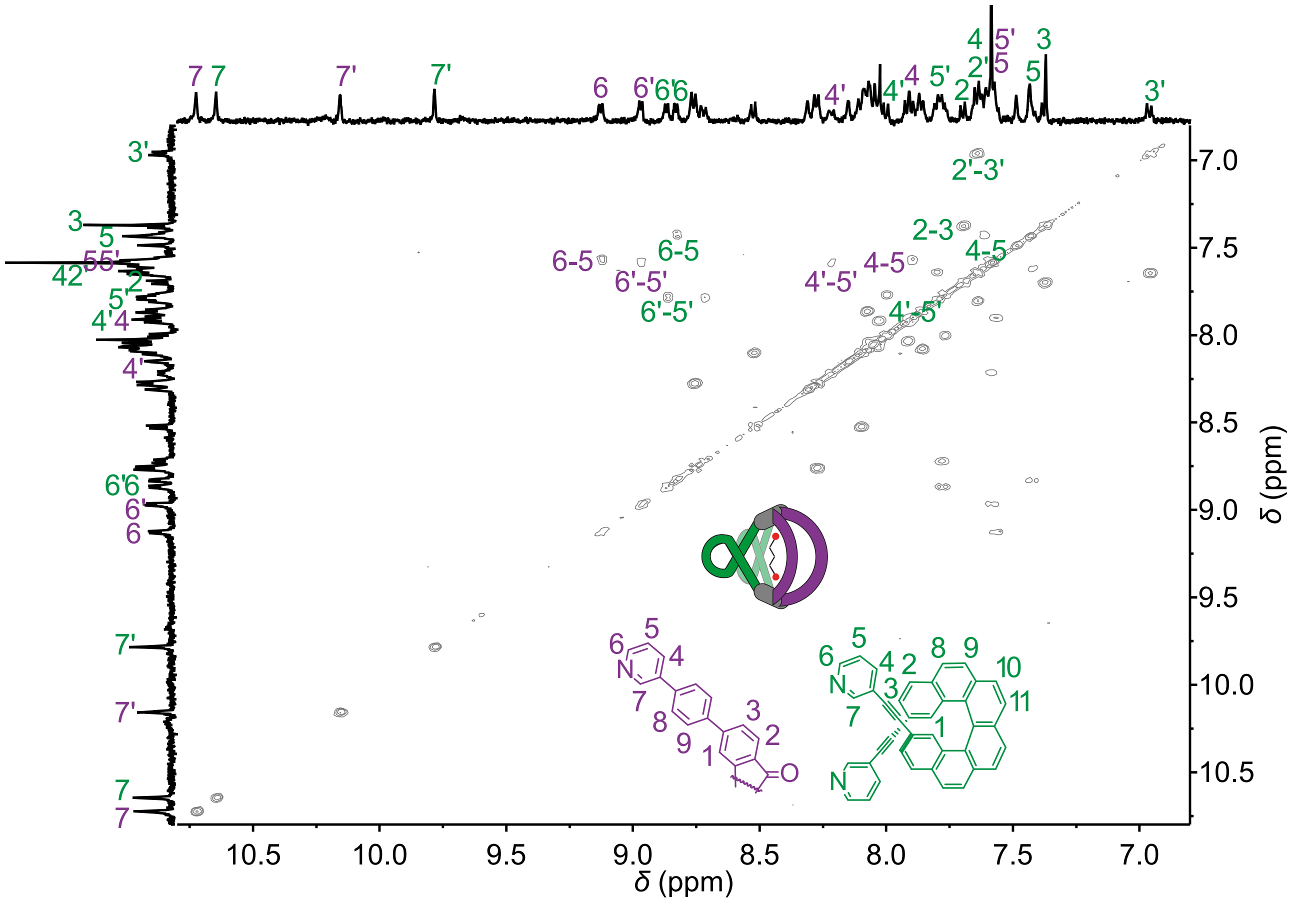

Figure S18. Partial $^{1}H–^{1}H$ COSY spectrum (500 MHz, 298K, CD_3CN) of heteroleptic cage [**G@Pd$_2$B$_2$M$_2$**]$^{4+}$.

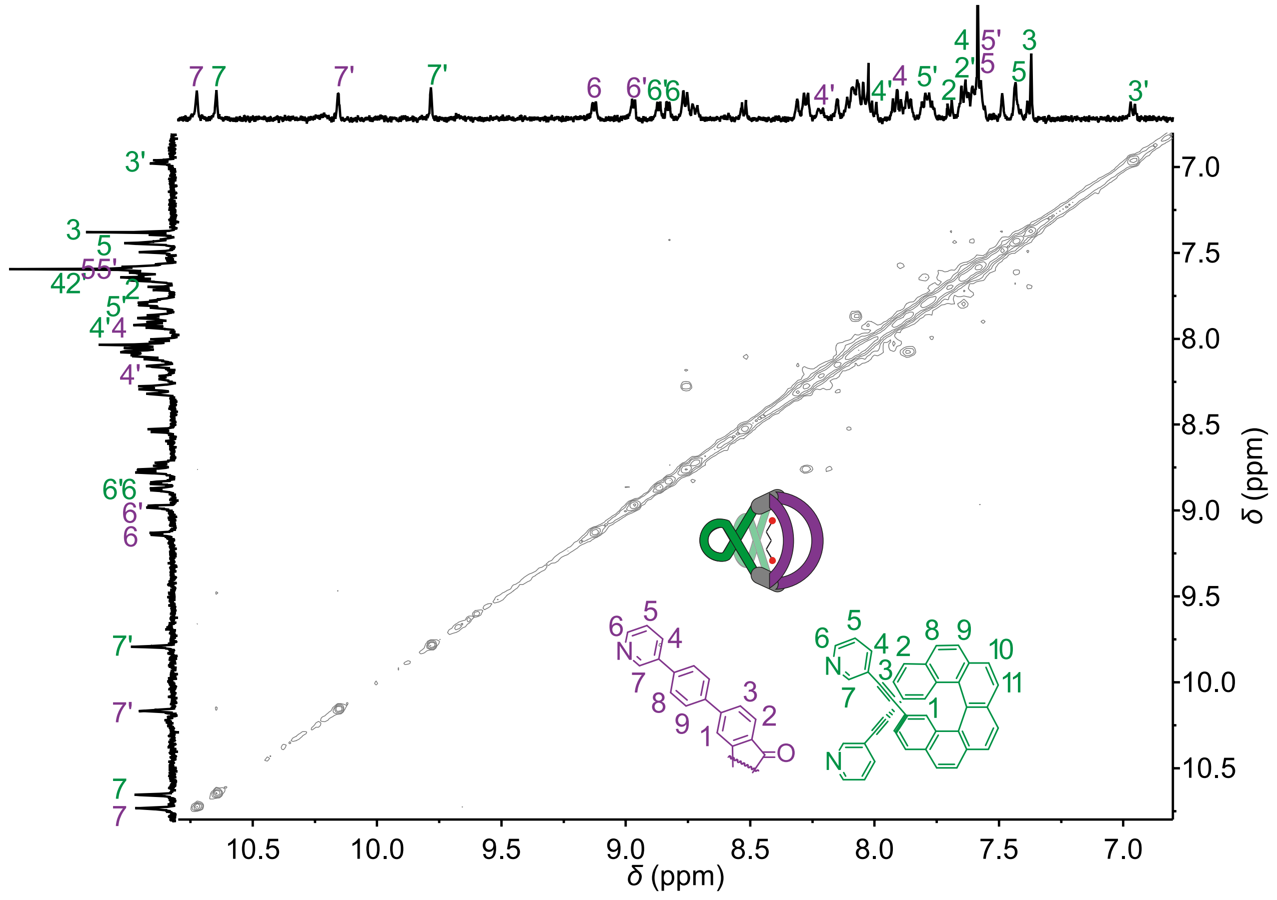

Figure S19. Partial $^{1}H–^{1}H$ NOESY spectrum (500 MHz, 298K, CD_3CN) of heteroleptic cage [**G@Pd$_2$B$_2$M$_2$**]$^{4+}$.

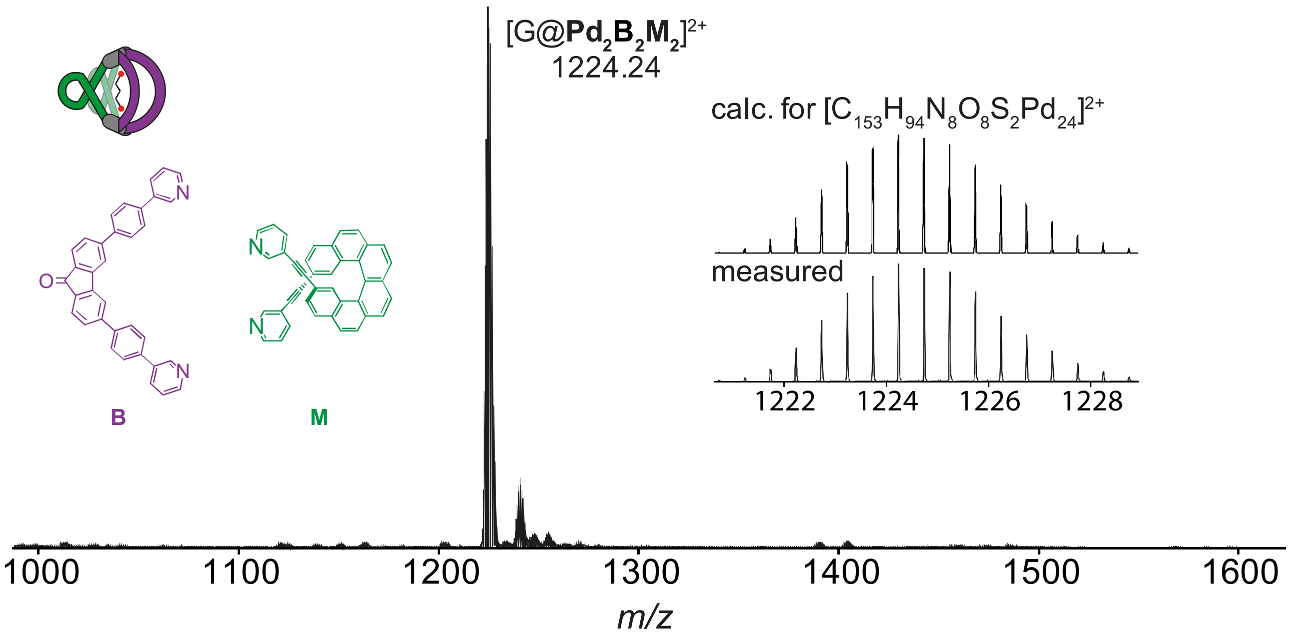

Figure S20. HR-ESI mass spectrum of **[G@Pd$_2$B$_2$M$_2$]$^{2+}$** (with 1 eq. of G).

3. Photophysical properties

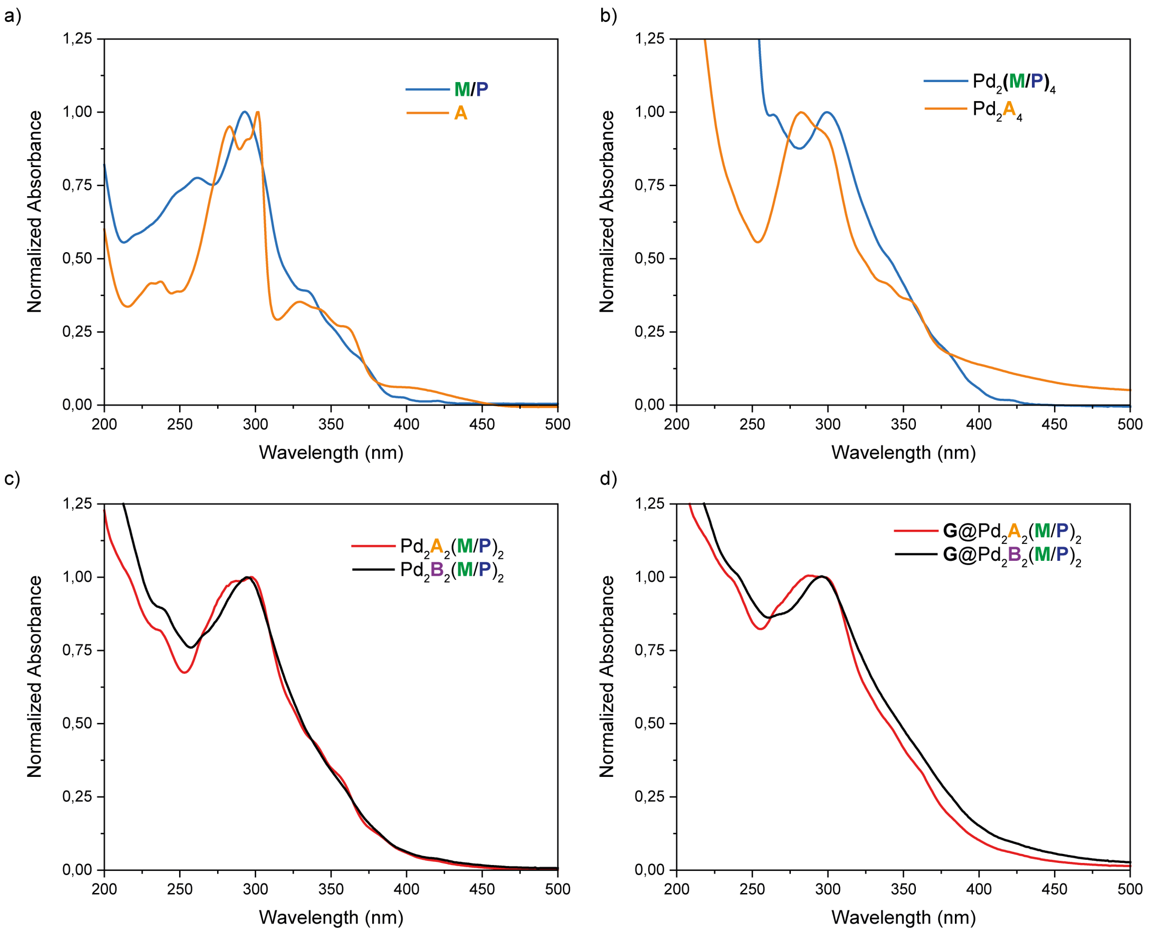

Figure S21. UV-Vis spectra (CD_3CN, RT) of a) ligands **A**, **M/P** (1×10^{-5} M); b) homoleptic cages Pd$_2$**A**$_4$ and Pd$_2$(**M/P**)$_4$, c) heteroleptic cages Pd$_2$**A**$_2$(**M/P**)$_2$ and Pd$_2$**B**$_2$(**M/P**)$_2$ and d) host-guest complexes **G**@Pd$_2$**A**$_2$(**M/P**)$_2$ and **G**@Pd$_2$**B**$_2$(**M/P**)$_2$ (3×10^{-5} M).

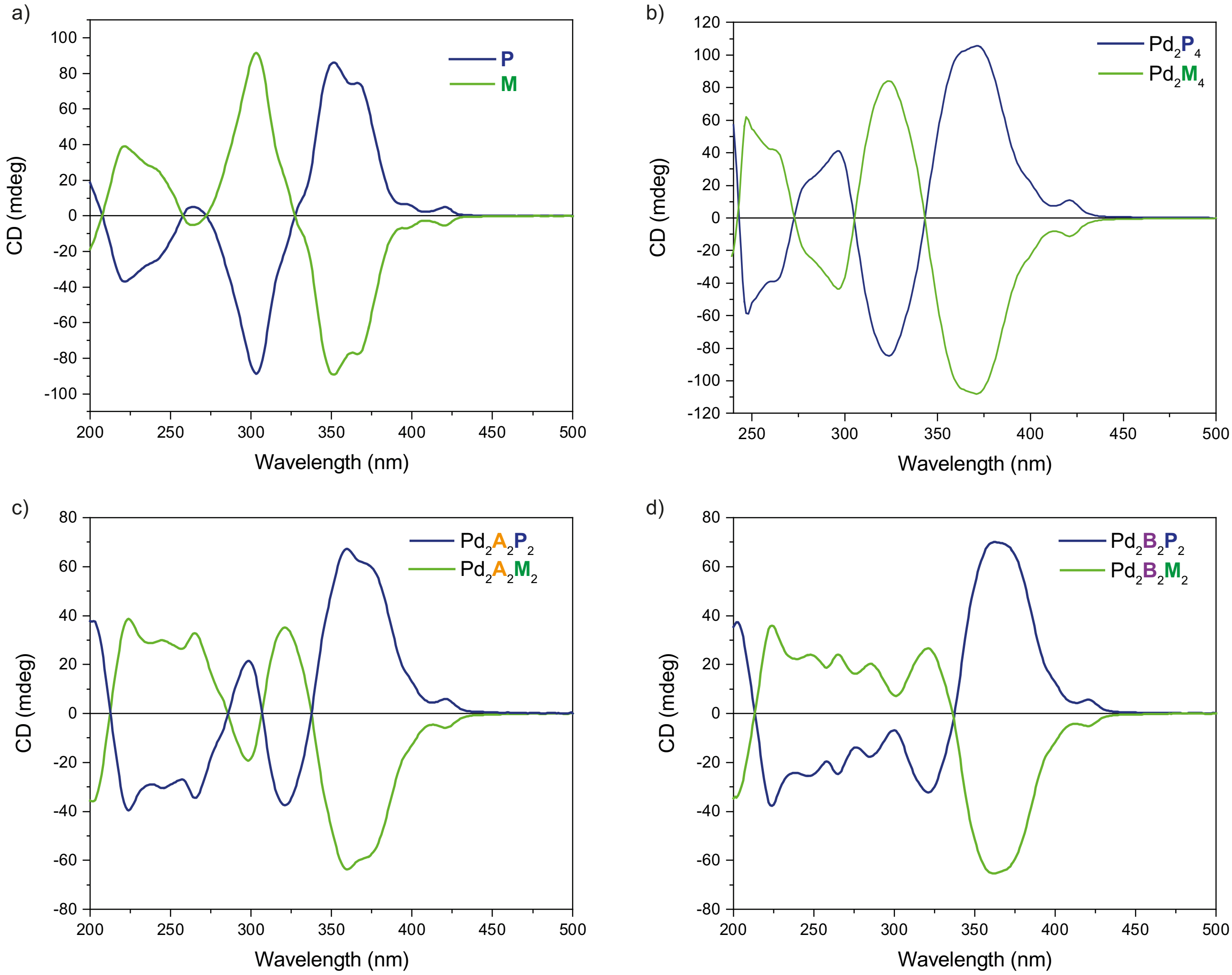

Figure S22. CD spectra (CD_3CN, RT) of a) ligands **M** and **P** (1×10^{-5} M); b) homoleptic cages Pd_2**M**$_4$ and Pd_2**P**$_4$ (diluted from DMF-d_7 stock solution), c) heteroleptic cages Pd_2**A**$_2$**M**$_2$ and Pd_2**A**$_2$**P**$_2$ and d) heteroleptic cages Pd_2**B**$_2$**M**$_2$ and Pd_2**B**$_2$**P**$_2$ (3×10^{-5} M).

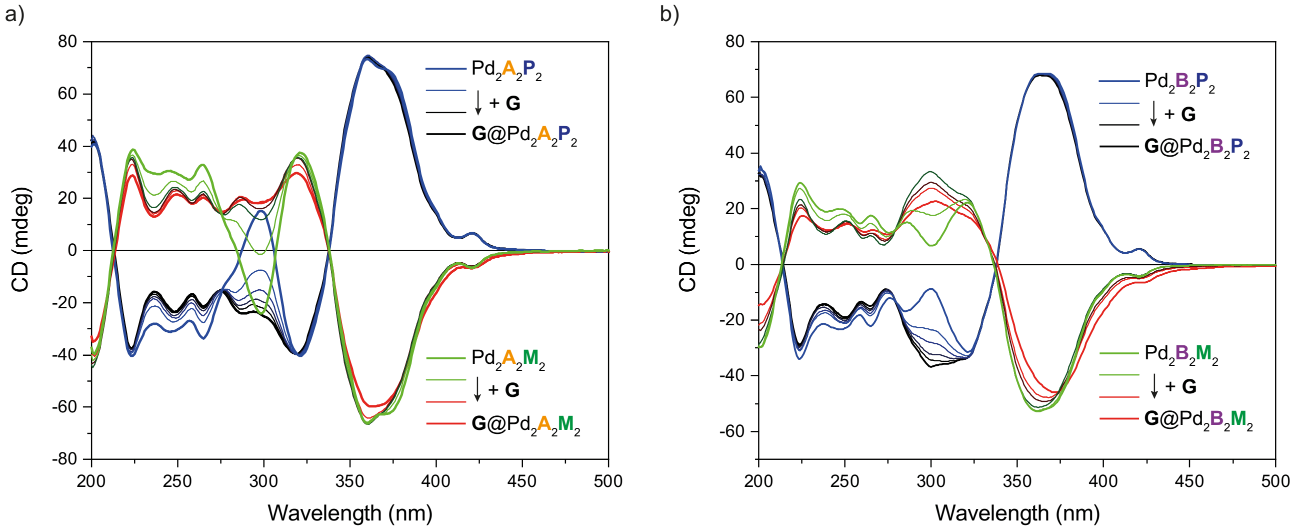

Figure S23. CD spectra (CD_3CN, RT) of guest addition to a) Pd_2**A**$_2$**M**$_2$ and Pd_2**A**$_2$**P**$_2$; b) Pd_2**B**$_2$**M**$_2$ and Pd_2**B**$_2$**P**$_2$ (3×10^{-5} M), steps of 0.2 eq of guest.

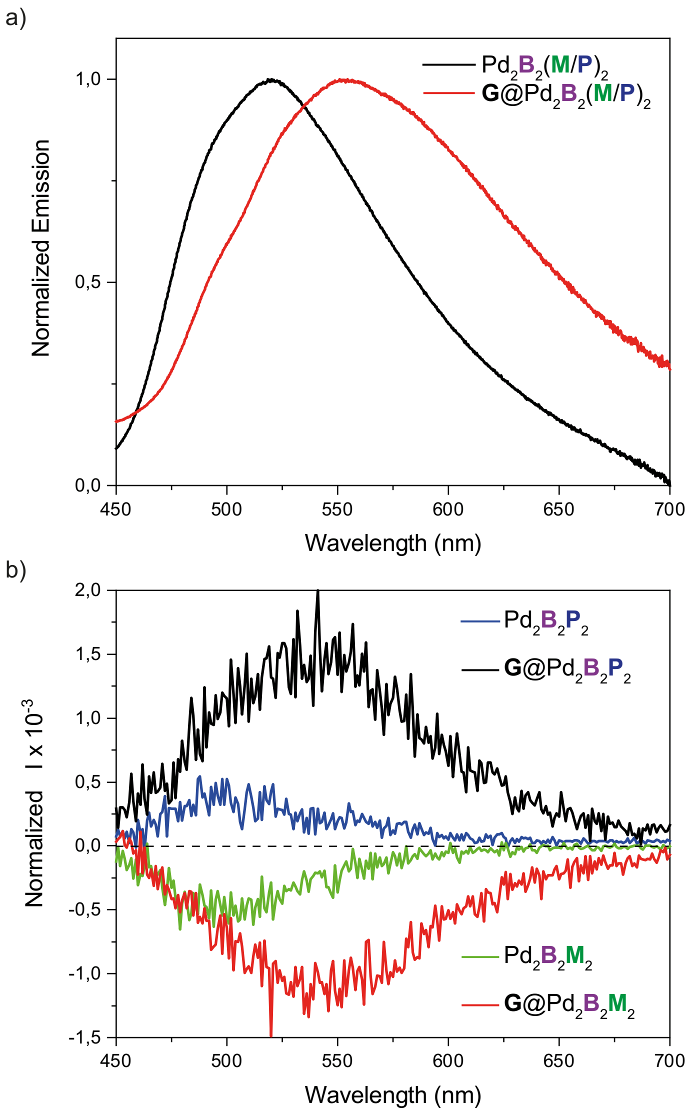

Figure S24. a) Fluorescence spectra of Pd_2**B_2**(**M/P**)$_2$, **G@**Pd_2**B_2**(**M/P**)$_2$ and b) CPL spectra of empty hosts Pd_2**B_2****M_2**, Pd_2**B_2****P_2** and host-guest complexes **G@**Pd_2**B_2****M_2** and **G@**Pd_2**B_2****P_2** (CD_3CN, RT, 3×10^{-5} M). λ_{ex} = 350 nm.

Table S1. Quantum Yield determination

	**A**	Pd_2**A_4**	Pd_2**A_2**(**M/P**)$_2$	**G@**Pd_2**A_2**(**M/P**)$_2$	Pd_2**B_2**(**M/P**)$_2$	**G@**Pd_2**B_2**(**M/P**)$_2$
QY (%)	6.1	2.9	1.5	1.1	2.8	1.4

λ_{ex} = 360 nm, CD_3CN, RT, concentrations chosen that the absorption at the excitation wavelength does not exceed 0.1, 3 mm quartz cuvette. Measured on a JASCO ILF-835 integrating sphere as accessory of the JASCO FP-8300 spectrofluorimeter. For each sample, 3-6 measurements were made and averaged.

4. Ion Mobility Mass Spectrometry

Ion mobility measurements were performed on a Bruker timsTOF instrument combining a trapped ion mobility (TIMS) with a time-of-flight (TOF) mass spectrometer in one instrument.

In contrast to the conventional drift tube method to determine mobility data, where ions are carried by an electric field through a stationary drift gas, the TIMS method is based on an electric field ramp to hold ions in place against a carrier gas pushing them in the direction of the analyzer. Consequently, larger sized ions that experience more carrier gas impacts leave the TIMS units first and smaller ions elute later. This method offers a much higher mobility resolution despite a smaller device size.

Briefly, in trapped ion mobility spectrometry (TIMS) the ions are held by an electric field gradient (EFG) while exposed to a constant N_2 flow. The source of the flow is a pressure difference (Δp) between the entrance and the exit of the TIMS-tunnel. Lowering the EFG results in the elution of the analyte according to its inverse mobility ($1/K_0$). Depending on the difference between start-EFG and end-EFG (ΔEFG) as well as the duration of this reduction (ramp time = t_r), rather high ion mobility resolutions can be achieved. Resolutions of 200 and higher was targeted in all the experiments using custom mode of measurement. In front of the trapping part of the TIMS-tunnel, a second ramp accumulates the ions for a given period of time (accumulation time = t_a) in order to enhance the overall duty cycle.

Since TIMS doesn't operate on first principles, a calibration is necessary. For calibration, Agilent Tune-Mix™ was used as purchased and the reported drift tube $^{DT}CCS_{N2}$ values published by Stow et al. have been used.[3] TIMS determines (calibrated) inverse mobilities which were converted to the tabulated $^{TIMS}CCS_{N2}$ values following Mason-Schamp equation:

$$\mathbf{CCS} = \left(\frac{3}{16N}\right)\left(\frac{2\pi}{kT}\right)^{0.5}\frac{q}{\sqrt{\mu}}\frac{1}{K_0}$$

where, q is the charge of the ion, N the number density of the collision gas, μ is the reduced mass of the ion and the N_2 collision gas, k the Boltzmann's constant, T the temperature in Kelvin. Both the analyte solution and the calibrants have been electrosprayed in cationic mode with the same experimental conditions. Therefore, the first part of the equation is constant for every ions measured in the same condition. The constant was found by calculating the reduced mobility of the calibrant ions and plotting them against their reported CCS. The plot was fitted using linear fit with fixed intercept at 0. The slope is the constant for the specific experiment. The $1/K_0$ found from the experiment was then multiplied as per the equation.

Optimized parameters: After the generation of ions by electrospray ionisation (ESI, analyte concentration: 0.1 mg/mL solvent: Acetonitrile, capillary voltage: 2000V, end plate offset voltage: 150 V, nebulizer gas pressure: 0.3 bar, dry gas flow rate: 3.0 l/min, dry temperature: 75 °C) the desired ions were orthogonally deflected into the TIMS cell consisting of an entrance funnel, the TIMS analyser (carrier gas: N_2, temperature: 305 K, entrance pressure: 2.72 mbar, exit pressure: 0.96 mbar, Δp = 1.77 mbar, t_r = 450 ms, t_a = 5 ms, a stepwise reduction of the electric field strength leads to a release of ion packages separated by their mobility. After a subsequent focusing, the separated ions are transferred to the TOF-analyser.[4-6]

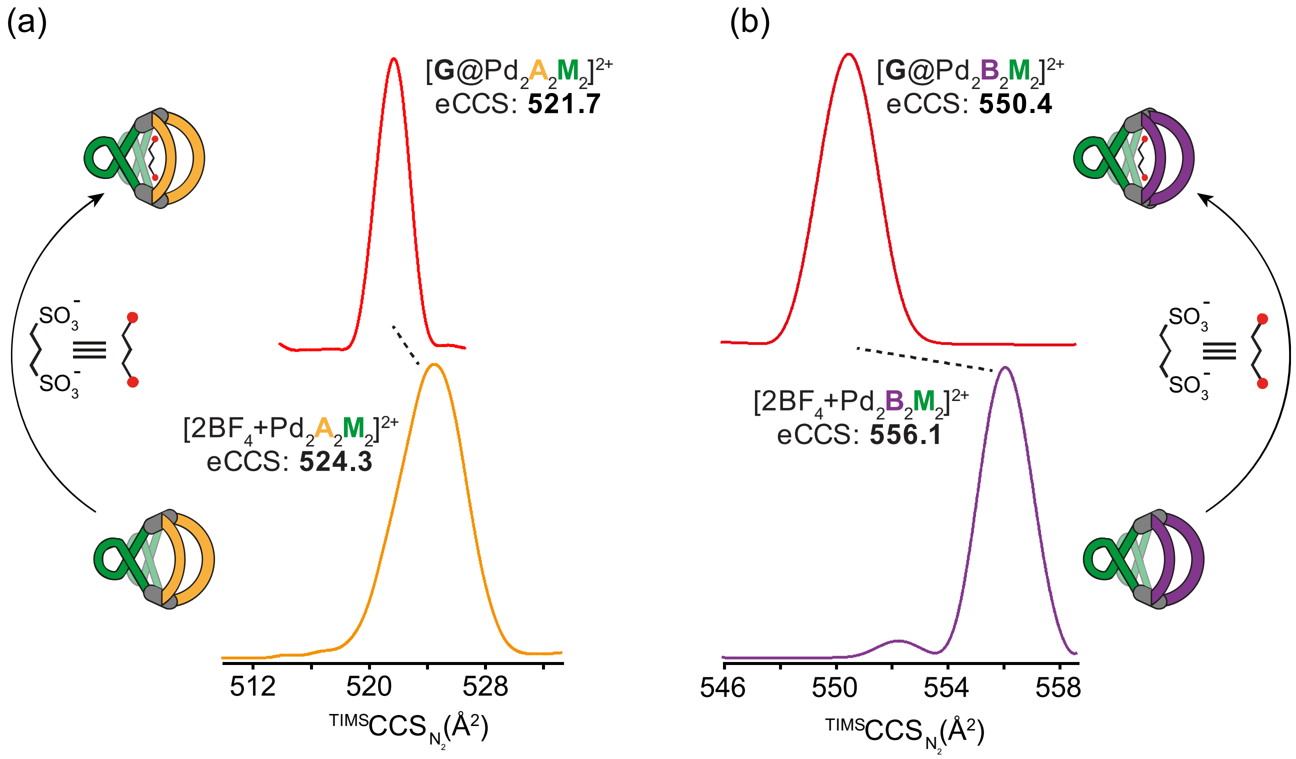

Figure S25. Collision cross sections obtained by trapped ion mobility ESI-TOF mass spectrometry of (**a**) heteroleptic $[2BF_4+Pd_2\mathbf{A}_2\mathbf{M}_2]^{2+}$ and $[\mathbf{G}@Pd_2\mathbf{A}_2\mathbf{M}_2]^{2+}$, (**b**) $[2BF_4+Pd_2\mathbf{B}_2\mathbf{M}_2]^{2+}$ and $[\mathbf{G}@Pd_2\mathbf{B}_2\mathbf{M}_2]^{2+}$. In contrast to the eCCS value of both empty heteroleptic cages (in orange and purple, respectively), after guest binding, both host-guest complex (in red) show a smaller eCCS value, indicating that guest binding occurred inside the cavity of cages.

5. Computational studies

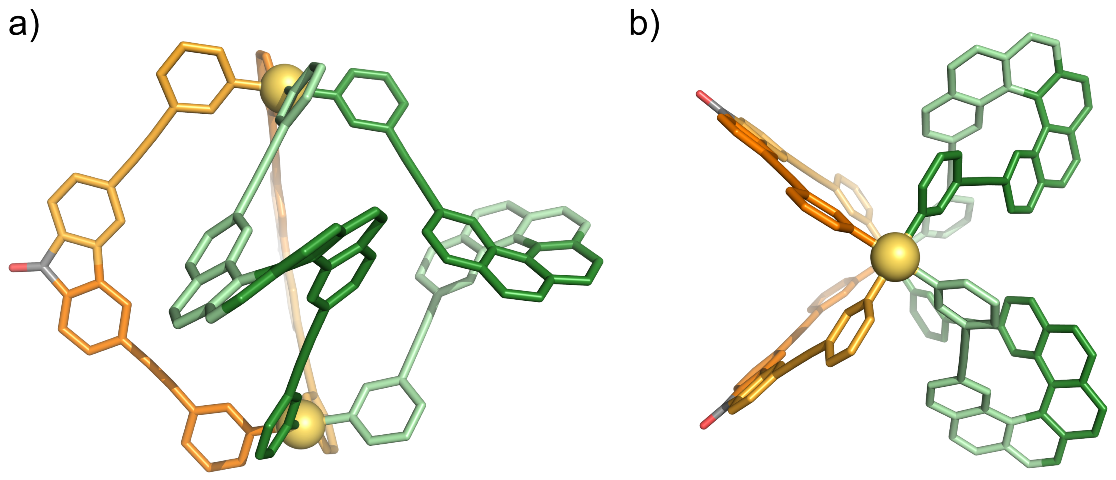

Figure S26. Model of heteroleptic cage Pd_2**A**$_2$**M**$_2$ optimized on ωb97xd/def2-SVP level in different views, a) side view, b) top view. Different chemical environments are indicated by different colours.

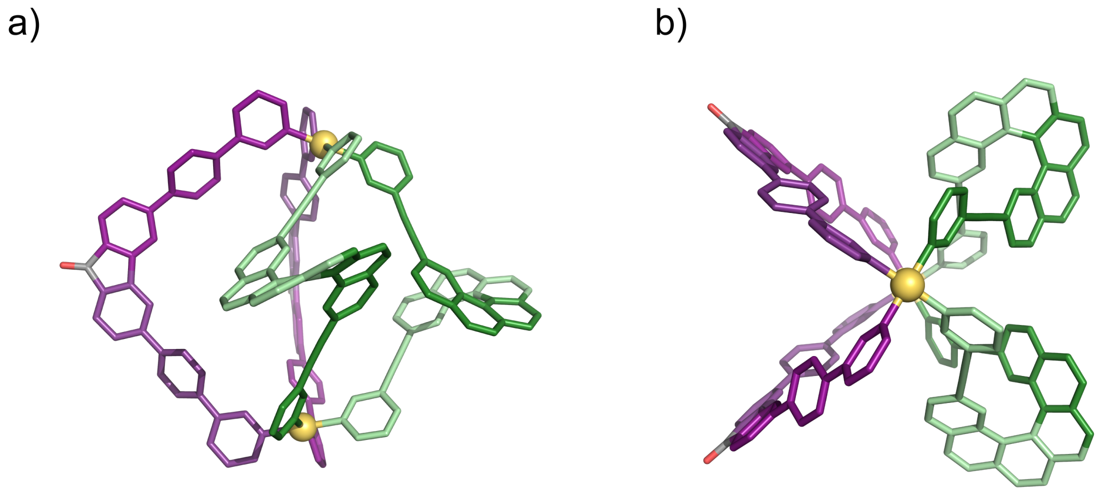

Figure S27. Model of heteroleptic cage Pd_2**B**$_2$**M**$_2$ optimized on ωb97xd/def2-SVP level in different views, a) side view, b) top view. Different chemical environments are indicated by different colours.

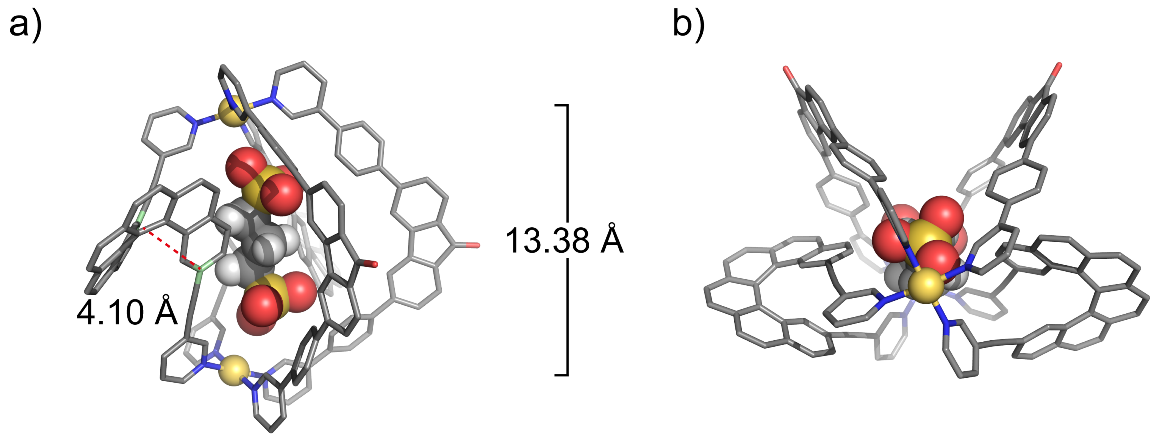

Figure S28. Model of ωb97xd/def2-SVP host-guest complex **G**@Pd_2**B**$_2$**M**$_2$ with highlight of helical pitch and Pd···Pd distances. a) side view, b) top view.

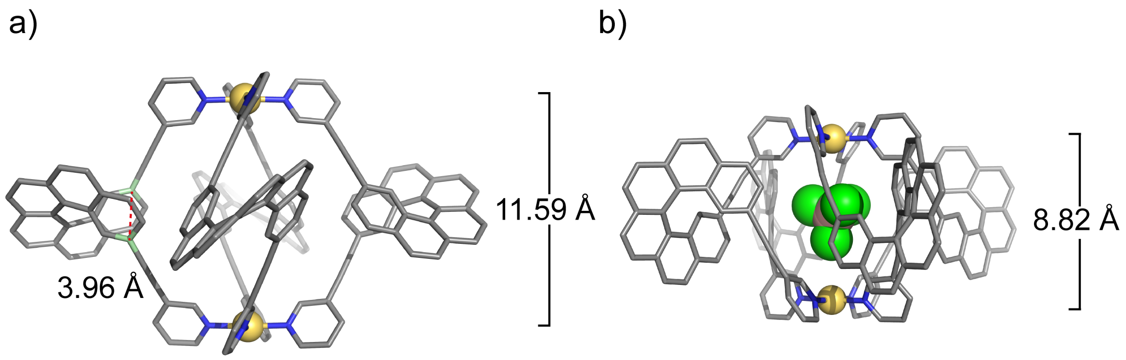

Figure S29. a) Anion-free, gas-phase model of homoleptic cage **Pd_2M_4** optimized on ωb97xd/def2-SVP level with highlight of helical pitch and Pd···Pd distance. b) crystal structure of **Pd_2M_4** with highlight of Pd···Pd distance. The encapsulated BF_4^- anion is shown in space-filling mode.

Structural analysis on DFT optimized (ωb97xd/def2-SVP) molecular structures

Cage	Pd_2M_4	$Pd_2A_2M_2$	$Pd_2B_2M_2$	G@$Pd_2A_2M_2$	G@$Pd_2B_2M_2$
Helical Pitch d (Å)	3.96	3.82	3.97	3.69 and 4.04	4.10
Pd···Pd d (Å)	11.593	12.319	13.63	12.50	13.38
g_{lum}	Not emitting	$\|0.9\times10^{-3}\|$	$\|0.4\times10^{-3}\|$	$\|3.5\times10^{-3}\|$	1.5×10^{-3} and -1.2×10^{-3}

Structural analysis on DFT optimized (B3LYP/def2-SVP) molecular structures

Cage	Pd_2M_4	$Pd_2A_2M_2$	$Pd_2B_2M_2$	G@$Pd_2A_2M_2$	G@$Pd_2B_2M_2$
Helical Pitch d (Å)	4.37	4.44	4.537	4.382- 4.423	4.477
Pd···Pd d (Å)	12.012	13.126	14.265	13.054	13.261
g_{lum}	Not emitting	$\|0.9\times10^{-3}\|$	$\|0.4\times10^{-3}\|$	$\|3.5\times10^{-3}\|$	1.5×10^{-3} and -1.2×10^{-3}

Energy comparison between tentative *cis*- and *trans*-heteroleptic cage isomers

	E *cis*-$Pd_2A_2M_2$ (Hartree)	E *trans*-$Pd_2A_2M_2$ (Hartree)	ΔE ($kJmol^{-1}$)
ωB97XD/def2-SVP	-5987.163945	-5987.162165	- 4.67
B3LYP/def2-SVP	-5988.595905	-5988.589809	-16.01

DFT geometry optimizations were performed with and without dispersion correction contributions, using ωb97xd or B3LYP functionals and computed using the Gaussian 16 software.[7] This has been done since the flexibility of the systems, especially for the cage and the host-guest system involving ligand **A**, can potentially lead to several conformations of similar energies. In both computational approaches, the trend followed is the same, with an increase of the helicenes' helical pitch and of the Pd···Pd distances when moving from ligand **A** to ligand **B.** Guest encapsulation results in a decrease of the Pd···Pd distance, particularly pronounced for **G@**Pd_2**B_2M_2** and almost neglectable for **G@**Pd_2**A_2M_2**.

Calculation of ligand absorption and emission features:

A full theoretical treatment of the observed photophysical features for the herein reported, hundreds of atoms counting coordination cages and host-guest complexes will be part of an extensive computational project including MD-based sampling of host and host-guest co-conformations (requiring development of an adapted force-field, studies of solvent and counter ion effects and analysis of trajectories), Boltzmann-weighted extraction of lowest-energy structures, QM electronic structure calculations with a variety of methods (i.e. different DFT functionals and basis sets to ensure proper treating of Pd cations and intracage and host-guest non-covalent interactions) and excited state calculations (including vibronic effects) to elucidate the nature of the electronic transitions giving rise to the observed absorption and emission features. Here, we give some first insights based on a simplified analysis comprising the organic ligands, only, that already indicate key phenomena of the experimental observations.

1. As shown in Figure S21, the absorption features of the hetero- and homoleptic Pd(II)-coordination cages largely resemble the UV-Vis spectra of the constituting ligands (with the helicene absorption undergoing a slight redshift and a loss of fine structure in particular in case of the heteroleptic cages). Hence, we chose to base our preliminary analysis of photophysical features on the individual, non-coordinated ligands to large simplify the computational efforts here.

2. Full photophysical characterization of a close isomer of helicene ligand **M**/**P** (*para*- instead of *meta*-pyridyl) has been reported by Autschbach, Crassous and Favereau, including both experimental absorption, emission, CD and CPL spectra as well as computed electronic transitions.[8] Essentially all reported photophysical features of the there reported compound perfectly superimpose with ligand **M**/**P** in our system. In particular, the reported emission maxima of the free ligands perfectly match and so do the g_{lum} values (there: 2.7 x 10^{-2}, here 2.8 x 10^{-2}).

3. As for the fluorenone ligand **A**, we performed TD-DFT calculations to compute the absorption and emission features as follows: gas-phase geometry optimization on CAM-B3LYP/6-31G+g(d,p) level of theory using Gaussian '16[7], followed by frequency calculation to confirm optimization into a minimum on the PES (a conformer was chosen as starting structure with both pyridine N lone pairs pointing in a colinear fashion as they do in the assembled cage as shown below; computations on a conformer with pyridines turned outside confirmed almost identical energy and electronic transitions). Then, a TD-DFT computation was performed to calculate vertical excitations with linear response (nstates=6). The obtained absorption maxima match well with the experimental values, i.e. for the two most intense bands (exp.: 285 nm; calc.: 274 nm and exp.: 300 nm; calc.: 298 nm), while the broader band at longer wavelengths (exp.: 325-375 nm; calc.: 366 nm) is more shifted and lacks fine structure in the computed spectrum (see Fig. S21a). Next, we optimized the first excited state (nstates=6, root=1) on the same level of theory and analyzed the output for the lowest energy (assuming the compound shows regular Kasha behaviour) S_1-S_0 singlet emission (in accordance with the experimentally observed lack of sensitivity of the emission to the presence of oxygen), yielding an emission wavelength of 483 nm (vertical transition, no vibronic effects considered), matching well to the experimentally observed emission maximum of ligand **A** at 500 nm (Fig. 5a). Frontier orbital analysis of this transition reveals that the corresponding transition is – as expected for an aromatic ketone – between an excited state MO with π-character spanning the aromatic rings of the backbone and featuring a nodal plane concerning the C=O bond (higher energy; Fig. S30 right) and a π-MO also on the delocalized aromatic backbone system (lower energy; Fig. S30 left).

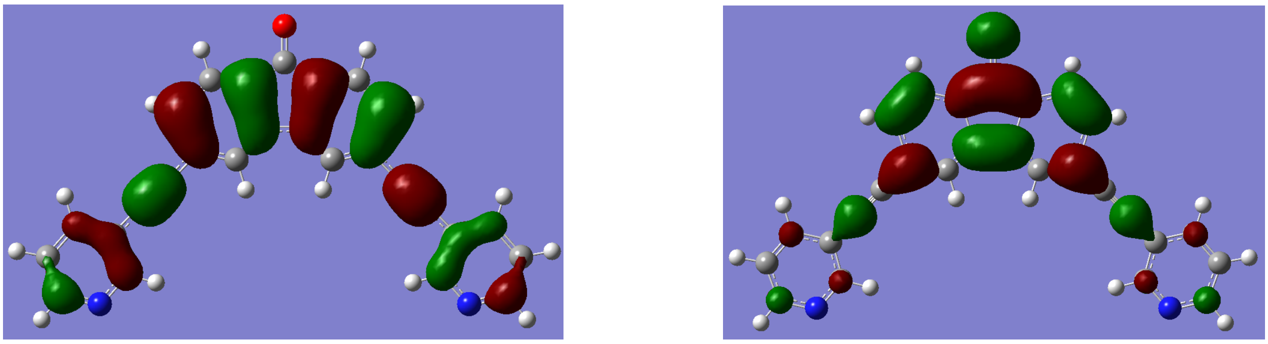

Figure S30. Left: HOMO of excited state geometry, right: LUMO of excited state geometry. Computed vertical transition at 483 nm ($S_1 \rightarrow S_0$, oscillator strength = 0.0435).

4. Next, the experimentally observed, strong emission red-shift upon addition of the guest was tried to be reproduced in the simplified computational model. Therefore, the excited state geometry of the ligand as computed above was combined with a model of the guest dianion (ground-state pre-optimized on same level of theory) in a geometry, that puts the guest's carbon and sulfur atoms in plane with the flat ligand with two sulfonate oxygen substituents pointing onto the ligand's pyridine H7 atoms (assignment see above) in a distance of 2.06 Å in accordance with the geometry optimized host-guest complex **G**@Pd$_2$**A**$_2$**M**$_2$ as shown in Fig. 3d. With this model, again TD-DFT calculations of vertical transitions were performed and the lowest wavelength transition, in accordance with Kasha's rule, was found to comprise orbitals of the same geometry as for the free ligand (compare Figs. S30 and S31) and feature a red-shifted emission at 507 nm, hence qualitatively supporting the experimental result (Fig. S32; exp. red-shift: 50 nm, calc.: 24 nm; computed transitions involving pure guest-localized MO contributions were excluded from the analysis as those would represent guest-ligand charge transfer states not considered relevant here).

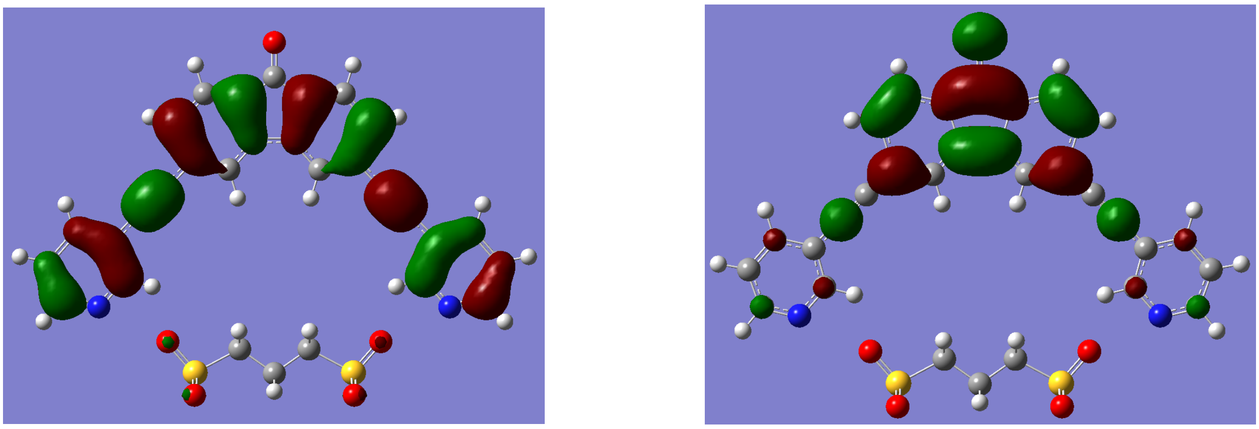

Figure S31. Left: HOMO of excited state geometry, right: LUMO of excited state geometry. Computed vertical transition at 507 nm ($S_1 \rightarrow S_0$, oscillator strength = 0.0757).

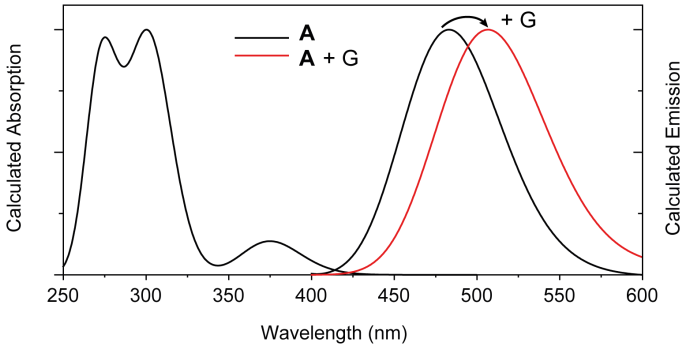

Figure S32. Calculated absorptions (left) and emission of ligand **A** and **A** in complex with guest **G**.

5. The question may be raised whether direct electronic interaction (e.g. strong exciton coupling or ligand-to-ligand FRET processes) between the chiral helicenes and the emitting fluorenones play a role in the emission and CPL of the heteroleptic cages and host-guest complexes. While only deeper photophysical characterization studies involving transient absorption and chiroptical analyses together with full-fletched MD/QM treatment of the entire coordination cage assemblies may serve to fully answer this, a preliminary experiment involving a chiral ligand **X** with an aliphatic backbone (not shown) that shows no absorption > 300 nm and cleanly forms a Pd$_2$**A**$_2$**X**$_2$ heteroleptic cage was found to exhibit analogous chirality transfer and CPL emission from the fluorenone ligand (excitation at 335 nm), indicating that strong electronic interaction between the helicenes and the fluorenones does not seem to be a requirement for the herein described phenomena.

In conclusion, our preliminary computational study on ligand **A** and the guest-ligand combination is able to reproduce the experimental absorption and emission features ascribed to ligand **A** as well as the red-shifting influence of the bound guest on the emission from the fluorenone (noteworthy is the very strong NMR downfield shift of protons H7 likewise pointing to a close and strong interaction between the sulfonate guest and the pyridine proton of ligand **A** in the host-guest assembly). In our current hypothesis, the helicene ligand does not play a significant electronic role in the observed chirality transfer, emission, CPL and guest modulation of the latter, but rather imprints its chirality onto the emissive moiety of the heteroleptic assembly via a structural influence on the overall geometry.

6. X-ray Crystallography

Table S2. Crystallographic data of [Pd_2**M**$_4$](BF_4)$_4$.

Compound	**[Pd_2M_4](BF_4)$_4$**
Identification code	kw99i_sq
CCDC number	2155501
Empirical formula	$C_{160}H_{88}B_2F_8N_8Pd_2$
Formula weight	2508.80
Temperature [K]	100(2)
Crystal system	triclinic
Space group (number)	$P1$ (1)
a [Å]	16.457(6)
b [Å]	17.718(6)
c [Å]	17.729(6)
α [°]	90.259(12)
β [°]	112.644(12)
γ [°]	105.872(13)
Volume [Å3]	4554(3)
Z	1
Density (calc.) (g/mL)	0.915
Absorption coefficient (mm-1)	1.983
F(000)	1278
Crystal size (mm3)	0.100×0.100×0.100
Crystal colour	yellow
Crystal shape	block
Radiation	CuK_α (λ=1.54178 Å)
2θ range for data collection (º)	5.23 to 73.08 (1.29 Å)
Reflections collected	35928
Independent reflections	8663 [0.0833]
Completeness (6.538°)	99.2 %
Data / restraints / parameters	8663/3591/1622
Goodness-of-fit on F2	0.855
R_1 [I>2σ(I)]	0.0648
wR_2 (all data)	0.2192
Largest peak/hole [eÅ^{-3}]	0.33/-0.22
Flack X parameter	0.20(3)

6.1. Crystal structure of [Pd_2M_4]$(BF_4)_4$

Yellow block-shaped crystals of [Pd_2**M**$_4$]$(BF_4)_4$ were grown by slow vapor diffusion of Et_2O into a solution of the cage in CD_3CN. Data was collected in-house on a Bruker D8 venture diffractometer equipped with an INCOATEC microfocus sealed tube (Iµs 3.0) using CuK_α radiation at 100 K. The data was integrated with APEX3 and the structure was solved by intrinsic phasing/direct methods using SHELXT[9] and refined with SHELXL[10] for full-matrix least-squares routines on F^2 and ShelXle[11] as a graphical user interface.

6.1.1. Specific refinement details.

Stereochemical restraints for the ligands **M** were generated by the GRADE program using the GRADE Web Server (http://grade.globalphasing.org) and applied in the refinement. A GRADE dictionary for SHELXL contains target values and standard deviations for 1,2-distances (DFIX) and 1,3-distances (DANG), as well as restraints for planar groups (FLAT). All displacements for non-hydrogen atoms were refined anisotropically. The refinement of ADP's for carbon, nitrogen and oxygen atoms was enabled by a combination of similarity restraints (SIMU) and rigid bond restraints (RIGU). The contribution of the electron density from disordered counterions and solvent molecules, which could not be modeled with discrete atomic positions were handled using the SQUEEZE routine in PLATON.[12, 13] The solvent mask file (.fab) computed by PLATON was included in the SHELXL refinement via the ABIN instruction leaving the measured intensities untouched.

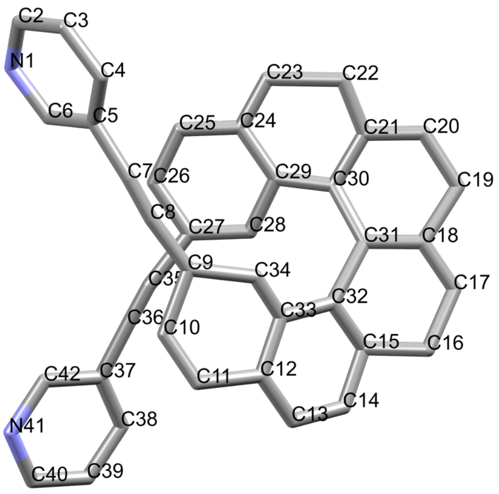

Figure S33. Atomic numbering scheme of residue MSH (ligand **M**).

7. References

[1] K. E. Ebbert, L. Schneider, A. Platzek, C. Drechsler, B. Chen, R. Rudolf, G. H. Clever, *Dalton Trans.* **2019**, *48*, 11070-11075.

[2] T. R. Schulte, J. J. Holstein, G. H. Clever, *Angew. Chem., Int. Ed.* **2019**, *58*, 5562-5566.

[3] S. M. Stow, T. J. Causon, X. Zheng, R. T. Kurulugama, T. Mairinger, J. C. May, E. E. Rennie, E. S. Baker, R. D. Smith, J. A. McLean, S. Hann, J. C. Fjeldsted, *Anal. Chem.* **2017**, *89*, 9048-9055.

[4] F. A. Fernandez-Lima, D. A. Kaplan, M. A. Park, *Rev. Sci. Instrum.* **2011**, *82*, 126106.

[5] J.-F. Greisch, J. Chmela, M. E. Harding, D. Wunderlich, B. Schäfer, M. Ruben, W. Klopper, D. Schooss, M. M. Kappes, *Phys. Chem. Chem. Phys.* **2017**, *19*, 6105-6112.

[6] D. R. Hernandez, J. D. DeBord, M. E. Ridgeway, D. A. Kaplan, M. A. Park, F. Fernandez-Lima, *Analyst* **2014**, *139*, 1913-1921.

[7] M. J. Frisch *et al*., Gaussian 16, Revision C. Gaussian, Inc., Wallingford CT, **2016**.

[8] K. Dhbaibi, L. Abella, S. Meunier-Della-Gatta, T. Roisnel, N. Vanthuyne, B. Jamoussi, G. Pieters, B. Racine, E. Quesnel, J. Autschbach, J. Crassous, L. Favereau, *Chem. Sci.* **2021**, *12*, 5522-5533.

[9] G. Sheldrick, *Acta Crystallogr. Sect. A* **2015**, *71*, 3-8.

[10] G. Sheldrick, *Acta Crystallogr. Sect. C* **2015**, *71*, 3-8.

[11] C. B. Hubschle, G. M. Sheldrick, B. Dittrich, *J. Appl. Crystallogr.* **2011**, *44*, 1281-1284.

[12] A. Spek, *J. Appl. Crystallogr.* **2003**, *36*, 7-13.

[13] A. Spek, *Acta Crystallographica Section D* **2009**, *65*, 148-155.
